# Polyketide-Terpene Hybrid Metabolites from an Endolichenic Fungus* Pestalotiopsis* sp.

**DOI:** 10.1155/2017/6961928

**Published:** 2017-05-16

**Authors:** Chao Yuan, Gang Ding, Hai-Ying Wang, Yu-Hua Guo, Hai Shang, Xiao-Jun Ma, Zhong-Mei Zou

**Affiliations:** ^1^Institute of Medicinal Plant Development, Yunnan Branch, Chinese Academy of Medical Sciences and Peking Union Medical College, Jinghong 666100, China; ^2^Institute of Medicinal Plant Development, Chinese Academy of Medical Sciences and Peking Union Medical College, Beijing 100193, China; ^3^College of Life Sciences, Shandong Normal University, No. 88 East Wenhua Road, Jinan 250014, China

## Abstract

Five new polyketide-terpene hybrid metabolites (**1**–**5**) with highly functionalized groups, together with six known derivatives (**6**–**11**), were isolated from the endolichenic fungus* Pestalotiopsis* sp. Their structures were elucidated by extensive NMR experiments including ^1^H, ^13^C, HMQC, COSY, and HMBC. The relative configurations of the new compounds were determined by analysis of coupling constants and ROESY correlations. The absolute configurations especially the secondary alcohol at C-15 in** 1** and secondary alcohol at C-14 in** 5** were established via the CD experiments of the in situ formed [Rh_2_(OCOCF_3_)_4_] complex with the acetonide derivatives. These compounds were tested for their inhibition activity against six plant pathogens. Compounds** 1** and** 5** exhibited pronounced efficiency against* Fusarium oxysporum*, and compounds** 5 **and** 6** potently inhibited* Fusarium gramineum* with MIC value of 8 *µ*g/mL, which revealed the plausible ecological role of endolichenic fungus in providing chemical protection for its host lichen in the fungus-plant relationship. The biosynthetic pathway of compounds** 1**–**11** was postulated for the first time, which paved the way for its further biosynthesis research.

## 1. Introduction

The highly prolific genus* Pestalotiopsis* often occurred on a broad range of substrata [1]. They could produce abundant diverse carbon skeleton compounds with many bioactivities [2]. Since discovery of the anticancer agent taxol from an endophytic fungal strain of the genus* Pestalotiopsis* sp. [3, 4], interest of bioactive compounds from this fungal genus has increased notably.

**Figure 1:**
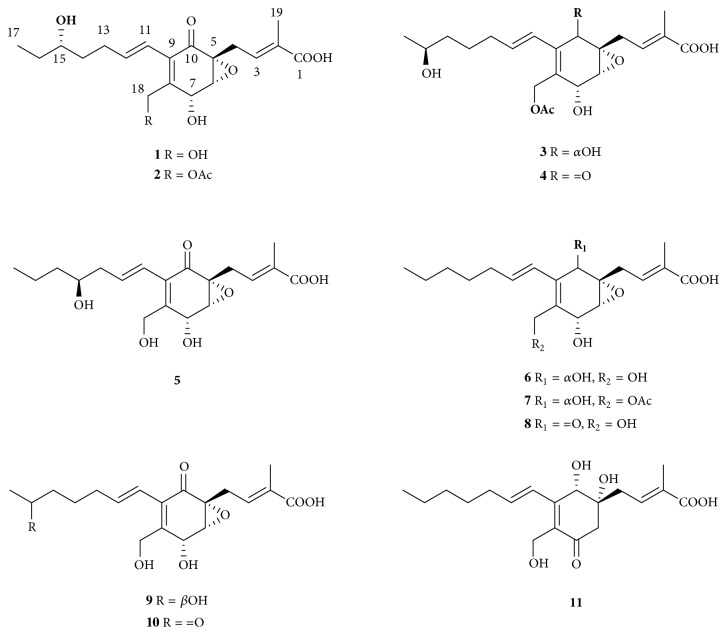
Chemical structures of compounds** 1**–**11**.

Endolichenic fungi inhabit the lichen thalli similarly to endophytes living in the intercellular spaces of healthy plant tissues [5]. Lichens are symbiotic organisms of photobiont and mycobiont. The photobiont provides the living space and nutrition for endolichenic fungi; in return, the endolichenic fungi might produce some secondary metabolites to help its host defend bio/abioattacks [6]. Chemical investigations revealed that endolichenic fungi could produce diverse secondary metabolites with significantly chemical ecological roles such as UV-protection, antiviral, and other biological activities [6–9]. Ambuic acid, a highly functionalized cyclohexenone metabolite with a wide range of biological activities, was first isolated from an endophytic fungus* Pestalotiopsis* spp. [10]. Later this bioactive compound was isolated from another two endophytic fungi* Pestalotiopsis* sp. [11, 12], which implied that ambuic acid and its analogs might be diagnostic metabolites in the chemical taxonomy of* Pestalotiopsis* sp. During our ongoing efforts to mine secondary metabolites from fungi inhabiting unique biotopes [13–16], an endolichenic fungus* Pestalotiopsis* sp. was targeted with purification of five new ambuic acid derivatives (**1**–**5**), together with six known ones (**6**–**11**) (Figure 1). Details of the isolation, structural elucidation, and bioactivity evaluation were reported herein.

## 2. Materials and Methods

### 2.1. Instruments

Optical rotations were obtained on a PerkinElmer 241 Polarimeter (Waltham, MA, USA), and CD measurements were performed with a JASCO J-815 Spectropolarimeter. UV data were measured using a Beckman Coulter DU 800 spectrometer (Tokyo, Japan). IR spectra were recorded using a Shimadzu FTIR-8400S spectrophotometer (Tokyo, Japan). NMR spectra were acquired with a Bruker 500 (Munich, Germany) instruments operating at 500 (^1^H) and 125 (^13^C) MHz using the solvent signals (CD_3_OD: *δ*_H_ 3.31/*δ*_C_ 49.0 ppm) as references. The HRESIMS experiments were carried out on a TOF-ESI-MS (Waters Synapt G2, USA) equipment. HPLC for purifications were performed on a Waters 2489 Semiprep-HPLC System using an ODS column (RP-18, 250 × 10 mm, YMC Pack, 5 *μ*m; detector: UV; Kyoto, Japan). Sephadex LH-20 (Pharmacia Biotech AB, Uppsala, Sweden) and silica gel (80–100, 100–200, and 200–300 mesh; Qingdao Marine Chemical Plant, Qingdao, China) were used for column chromatography, and thin layer chromatography (TLC) was carried out with glass precoated silica gel GF254 (0.20–0.25 mm; Qingdao Marine Chemical Plant, Qingdao, China).

### 2.2. Fungal Material

An endolichenic fungus* Pestalotiopsis* sp. was isolated from the lichen* Cetraria islandica* (L.) Ach. collected from Laojun Mountain, Yunnan Province, China, and was identified by one of the coauthors (Hai-ying Wang) based on the nuclear rDNA ITS sequences (GenBank: KY275424). The fungal strain was cultured on slants of potato dextrose agar (PDA) at 25°C for 10 days. To prepare the seed culture, the agar plugs were added to three 250 mL flasks, each containing 50 mL sterile potato dextrose broth (PDB) media. Flask cultures were incubated at 25°C on a rotary shaker at 170 rpm for 5 days. Large scale cultivation was carried out in twenty 500 mL Fernbach flasks each containing 60 g of rice and 90 mL of water. Each flask was inoculated with 5 mL of the spore inoculums and incubated at room temperature for 40 days.

### 2.3. Extraction and Isolation

The culture was extracted with EtOAc three times, and the organic solvent was evaporated under vacuum to yield the crude extract (17.0 g), which was subjected to silica gel column chromatography (CC) eluting with CH_2_Cl_2_-CH_3_OH (100 : 0, 100 : 1, 100 : 2, 100 : 3, and 100 : 5) to afford five fractions (A–E). Fraction C (1.5 g), eluting with 100 : 2 CH_2_Cl_2_-CH_3_OH, was separated with Sephadex LH-20 column chromatography using MeOH as eluent to give two subfractions. Compound** 8** (52.0 mg) was recrystallized from subfraction 1 with MeOH, and subfraction 2 was subjected to semipreparative reversed phase HPLC (35–100% MeOH in 40 min; 2 mL/min) to afford** 10 **(2.5 mg; *t*_R_ 20.4 min). Fraction D (56.0 mg) was further separated by Sephadex LH-20 CC using CH_3_OH as eluent to afford two subfractions. Subfraction 1 was further purified by semipreparative reversed phase HPLC (28–50% MeCN in 30 min; 2 mL/min) to afford** 4 **(2.0 mg; *t*_R_ 17.7 min) and** 2 **(3.0 mg; *t*_R_ 19.3 min). Subfraction 2 was purified by semipreparative RP HPLC (47–75% MeCN in 30 min; 2 mL/min) to yield** 7 **(5.0 mg; *t*_R_ 15.7 min). The fraction E (200 mg) eluting with 100 : 5 CH_2_Cl_2_-CH_3_OH was separated by Sephadex LH-20 CC eluted with CH_3_OH to afford two subfractions. One subfraction (110.3 mg) was purified by HPLC (35% MeOH in H_2_O for 2 min, followed by 35–100% MeOH for 42 min; 2 mL/min) to afford** 3** (6.0 mg, *t*_R_ 17.5 min),** 9** (7.0 mg, *t*_R_ 13.0 min),** 1** (11.0 mg, *t*_R_ 14.2 min),** 5** (13.0 mg, *t*_R_ 16.0 min), and** 11 **(21.0 mg, *t*_R_ 21.6 min), and the other subfraction was subjected to reversed phase HPLC (28% MeOH in H_2_O for 2 min, followed by 28–50% MeOH for 30 min; 2 mL/min) to afford** 6 **(12.0 mg; *t*_R_ 24.5 min).

### 2.4. Absolute Configuration of the Secondary Alcohol in** 1** and** 5** [17, 18]

Compound** 1** (2.0 mg) was treated with 2,2-dimethyoxypropane (1.0 mL) and pyridinium p-toluene sulfonate (1.0 mg) and then stirred at 30°C for 5 h under N_2_ circumstance [19]. The reaction solution was evaporated in vacuum and purified by semipreparative reversed phase HPLC (50–100% CH_3_CN in H_2_O for 30 min; 2 mL/min) to yield the acetonide** 1a** (1.0 mg, *t*_R_ 22.3 min); for ^1^H NMR spectrum see Figure S32, in Supplementary Material available online at https://doi.org/10.1155/2017/6961928.

Compound** 5** (2.0 mg) was treated the same way as** 1** to yield the acetonide** 5a** (0.5 mg, *t*_R_ 19.8 min) by semipreparative HPLC (50-100% MeCN in H_2_O in 30 min, 2 mL/min); for ^1^H NMR spectrum see Figure S33.

A sample of** 1a** (0.5 mg, or** 5a**) was dissolved in a dry solution of the stock [Rh_2_(OCOCF_3_)_4_] complex (1.5 mg) in CH_2_Cl_2_ (300 *µ*L) and was subjected to CD measurements. The first CD spectrum was recorded immediately after mixing and its time evolution was monitored until being stationary (ca. 10 min after mixing). The inherent CD was subtracted. The observed sign of the E band at around 322 nm in the induced CD spectrum was correlated to the absolute configuration of the C-15 (or C-14) secondary alcohol moiety.

### 2.5. Cytotoxicity Bioassay

All the isolates were tested for their cytotoxicity against three human cancer cell lines A549, HepG 2, and Hela. The cancer cells were incubated in DMEM medium (HyClone, USA), added with 10% fetal bovine serum (HyClone, USA), and cultured in 5% CO_2_ incubator at 37°C. The cytotoxicity tests were performed according to the MTT (3-(4,5-dimethylthiazol-2-yl)-2,5-diphenyl tetrazolium bromide) method [20], using etoposide as positive control.

### 2.6. Antibacterial Assay

A serial dilution method was employed for the determination of the antibacterial activities of the samples in triplicate, according to the National Center for Clinical Laboratory Standards (NCCLS) recommendations [21]. Three bacterial strains* Staphylococcus aureus* subsp. aureus (DSM 799),* Escherichia coli* (DSM 1116), and* Bacillus subtilis* (DSM 1088) were donated by Gang Li in School of Pharmacy, Qingdao University. Targeted microbes were prepared in MHB (Mueller-Hinton Broth) cultures, respectively, and the final spore suspensions of bacteria were 10^6^ CFU/mL. Test compounds were transferred to 96-well clear plate, with DMSO solvent no more than 1% of the total volume. The suspensions of test organisms were added to each well, achieving a final volume of 100 *µ*L (streptomycin was used as positive control). After incubation (37°C for 24 h), the absorbance at 595 nm was measured using a microtiter plate reader. The inhibition rate was measured and finally afforded the MIC values.

### 2.7. Antifungal Assay

Antifungal bioassays were also performed following the NCCLS recommendations, and six phytopathogenic fungi* Botrytis cinerea* (ACCC 37347),* Verticillium dahliae* (ACCC 36916),* Fusarium oxysporum* (ACCC 37438),* Alternaria solani* (ACCC 36023),* Fusarium gramineum* (ACCC 36249), and* Rhizoctonia solani* (ACCC 36124) were obtained from Agricultural Culture Collection of China (ACCC). Test microbes were cultured in Potato Dextrose Broth (PDB) and obtained the test concentrations of 10^4^ hyphae/mL. Test suspensions were added to each well achieving a final volume of 100 *µ*L. Test compounds (dissolved in DMSO following serial dilutions) were transferred into each well (ketoconazole as positive control); after incubation (28°C for 48 h), alamarBlue (10 *µ*L of 10% solution) was added to each well as an indicator; then the fluorescence intensity was measured at Ex/Em = 544/590 nm. The inhibition rate was calculated to afford MIC values. All the bioassays were performed in triplicate.

## 3. Results and Discussion

### 3.1. Structural Determinations of Isolated Compounds

**Figure 2:**
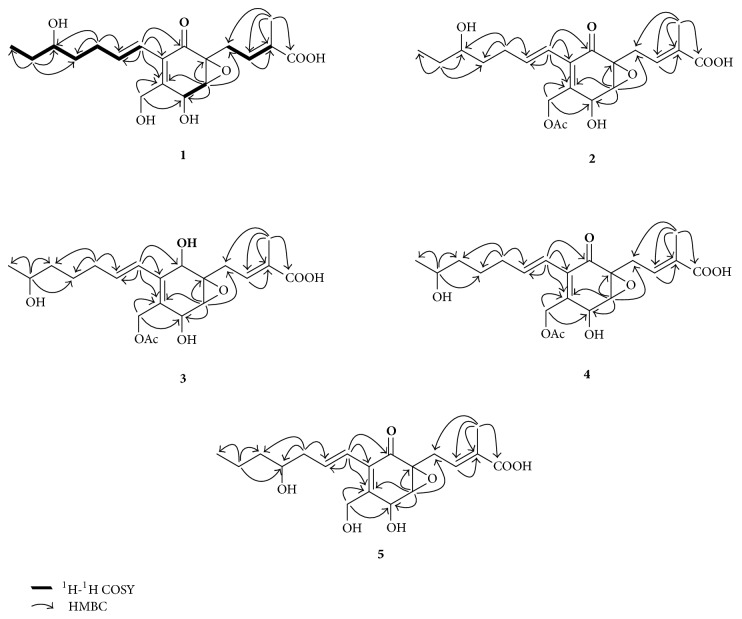
^1^H-^1^H COSY and key HMBC correlations of compounds** 1**–**5**.

Compound** 1** was isolated as brown oil, [*α*]_D_^20^  −43.1 (*c* 0.12, methanol). Its molecular formula was determined to be C_19_H_26_O_7_ (seven degrees of unsaturation) on the basis of the pseudomolecular [M + Na]^+^ ion at *m*/*z* 389.1587 (calcd 389.1576) together with consideration of NMR data. Its 1D NMR (Table 1, Figures S1 and S2) and HSQC data (Figure S5) showed resonances for two methyl groups, five methylenes (one oxygenated), three* O*-methines, six olefinic carbons (three of which were protonated), one oxygenated* sp*^3^ quaternary carbon, one carboxylic carbon (*δ*_C_ 171.2, C-1), and one*α*,*β*-unsaturated ketone carbon (*δ*_C_ 196.0, C-10). These data accounted for all the NMR resonances for** 1**. The ^1^H-^1^H COSY NMR data (Figure S3) of** 1** showed the three isolated spin-systems of C-4-C-3-C-19 (allylic coupling between H-3 and CH_3_-19), C-6-C-7, and C-11-C-17. HMBC correlations (Figure S4) from H-4 to C-5, C-6, and C-10; from H-6 to C-5, C-7, and C-8; from H-18 to C-7, C-8, and C-9; from H-11 to C-8, C-9, and C-10 established one cyclohex-2-en-one moiety with the C-4, C-18, and C-11 attached to C-5, C-8, and C-9, respectively. HMBC correlations from H-3 to C-1 and C-19 and from H-19 to C-1, C-2, and C-3 suggested a methyl and a carboxylic group located at C-2 (Figure 2). With comparison of NMR data of C-5 (*δ*_C_ 61.2) and C-6 (*δ*_C_ 61.1) with those of ambuic acid and its analogs, it demonstrated that these two carbons must form an epoxide ring. Molecular weight and the chemical shifts of H-15 and CH_2_-18 implied that a hydroxyl group was attached at both carbons, respectively. On the basis of these data, the planar structure of** 1** was proposed.

**Figure 3:**
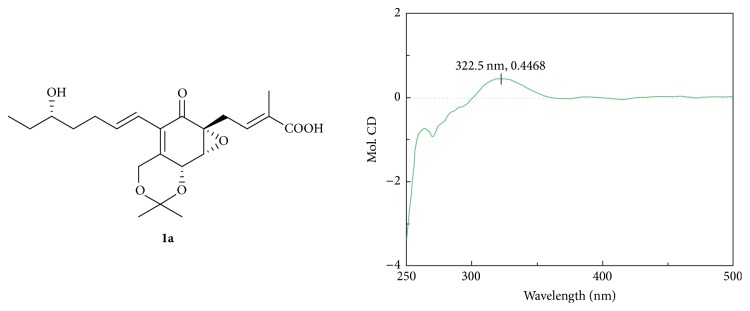
CD spectrum of Rh-complex of** 1a** with the inherent contributions subtracted.

The relative configurations of** 1** were deduced by the ^1^H-^1^H coupling constants and ROESY data (Figure S6). The C-11/C-12, C-2/C-3 double bonds were assigned* E*-geometry on the basis of the large coupling constant (*J* = 16.0 Hz) observed between H-11 and H-12 and ROESY correlation of CH_2_-4 with CH_3_-19. The small vicinal coupling constant (*J*_6, 7_ = 3.0 Hz) suggested a* cis* orient between H-6 and H-7; the ROESY correlation of H-6 with CH_2_-4 indicated these protons were on the same face of the cyclohex-2-en-one ring. The CD spectrum (Figure S7) of** 1** showed positive (350 nm) and negative (240 nm) Cotton effects, which were similar to those of ambuic acid derivatives [11], macrophorin A [22], and (+)-epoxydon [23], suggesting the 5*R*, 6*R*, and 7*R* in structure** 1**. The absolute configuration of the C-15 secondary alcohol in** 1** was deduced via the CD data of the in situ formed [Rh_2_(OCOCF_3_)_4_] complex with acetonide** 1a** (Figure 3). The Rh-complex of** 1a** displayed a positive Cotton effects at near 323 nm, suggesting the 15*S* absolute configuration [17].

Compound** 2** gave a molecular formula of C_21_H_28_O_8_ (eight degrees of unsaturation) by analysis of its HRESIMS (*m*/*z* 431.1684 [M + Na]^+^). The extra 42 mass units compared to that of** 1** suggested the presence of an acetyl group. Analysis of the 1D NMR spectroscopic data (Table 1) (Figures S8 and S9) and HSQC data (Figure S11) of** 2** revealed structural similarity to those of** 1**, except that the oxygenated methylene protons (CH_2_-18) were shifted downfield to *δ*_H_ 4.90 and 4.96, respectively, in** 2**. In addition, NMR resonances corresponding to an acetyl group (*δ*_H_ 2.06; *δ*_C_ 20.7 and 172.4) were observed, indicating that the hydroxyl group attached at C-18 was acylated. This hypothesis was confirmed by an HMBC correlation (Figure S10) from CH_2_-18 to the carboxyl carbon at *δ*_C_ 172.4. Thus the gross structure of compound** 2** was established as shown.

The relative configuration of** 2** was the same as that of** 1** by analysis of the ^1^H-^1^H coupling constants and ROESY data (Figure S12) of** 2**. The absolute configuration of C-5, C-6, and C-7 in** 2** was established on the basis of CD data. The CD spectrum of** 2** (Figure S13) showed positive (355 nm) and negative (243 nm) Cotton effects, indicating the 5*R*, 6*R*, and 7*R* configuration. From the view of biogenetic pathway together with considering the nearly same NMR data (C/H-15) as that of the coisolated analog** 1**, the absolute configuration of C-15 was postulated to be *S*.

The molecular formula of compound** 3** was determined to be C_21_H_30_O_8_ (seven degrees of unsaturation) on the basis of HRESIMS analysis (*m*/*z* 433.1848 [M + Na]^+^). Analysis of the ^1^H and ^13^C NMR (Table 1) (Figures S14 and S15) together with HSQC data (Figure S17) indicated that** 3** possess similar structure to that of the known compound (number 4 in the paper) reported by Qi and coworkers except that an additional hydroxyl group as a multiplet was found in** 3 **[12]. The HMBC correlations (Figure S16) confirmed that the hydroxy moiety was anchored at C-16. Thus the planar configuration of** 3** was determined.

The relative configurations for C-5, C-6, and C-7 in** 3** were deduced to be the same as those in** 1** by comparison of the coupling constants and ROESY data (Figure S18). In ROESY experiment of** 3**, the cross-peaks from H-10 to H-4, H-6, and H-7 suggested that H-10, H_2_-4, H-6, and H-7 were on the same face of the cyclohex-2-en-one ring. The CD spectrum of** 3** (Figure S19) showed negative Cotton effects in the regions of 238 nm the same as that of known analog [12]. Therefore, compound** 3** was deduced to have the 5*S*, 6*R*, 7*R*, and 10*S* except the C-16.

The molecular formula of compound** 4** was determined to be C_21_H_28_O_8_ (eight degrees of unsaturation) on the basis of HRESIMS analysis (*m*/*z* 431.1692 [M + Na]^+^) with less 2 mass units compared to that of** 3**, suggesting the presence of an additional unsaturation degree. Detailed analysis of the NMR spectra (Table 2) (Figures S20 and S21) together with HSQC data (Figure S23) especially the ^13^C NMR (C-8 and C-9) confirmed that the oxymethine (C-10) in** 3** must be oxidized to be the corresponding ketogroup in** 4**, which shaped *α*,*β*-unsaturated double bond leading to the chemical shift value of C-8 to be downfielded. This conclusion was further confirmed by HMBC correlations (Figure S22) from CH_2_-4, H-6, and H-11 to the ketogroup (*δ*_C_ 195.4, C-10).

The relative configurations of** 4** for C-5, C-6, and C-7 were determined in the same way with** 3** on the basis of the coupling constants and ROESY data (Figure S24). The CD data (S25) displayed the same Cotton curves as those** 1** and** 2**, which established the absolute configuration of** 4** as 5*R*, 6*R*, and 7*R*. Modified Mosher's reaction was tried to determine the absolute configuration of C-16 in** 3** and** 4**, but it was not successful. Thus consideration of biosynthetic pathway and nearly the same NMR data as those** 9** and** 10** at C-16 implied their same stereochemistry.

**Figure 4:**
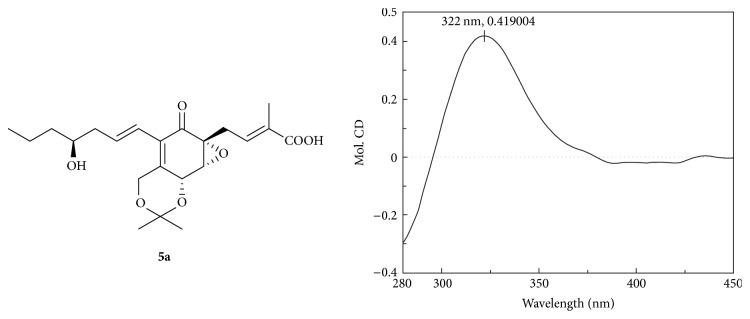
CD spectrum of Rh-complex of** 5a** with the inherent contributions subtracted.

Compound** 5** was assigned the molecular formula C_19_H_26_O_7_ (seven degrees of unsaturation) by HRESIMS (*m*/*z* = 389.1584 [M + Na]^+^). Comparison of the 1D NMR spectroscopic data (Figures S26 and S27) of** 5** with those of** 1** revealed that chemical shift values of H/C-15 (*δ*_H_ 3.64; *δ*_C_ 71.9) in** 1 **were transferred to *δ*_H_ 3.48 and *δ*_C_ 73.2, respectively, in** 5**, together with HSQC data (Figure S29), suggesting that the position of hydroxyl on aliphatic chain was changed in those two compounds. HMBC correlations (Figure S28) confirmed that C-14 possessed a free hydroxyl group. The relative configurations for C-5, C-6, and C-7 in** 5** were deduced to be the same as those in** 1** by comparison of the ^1^H-^1^H coupling constants and ROESY data (Figure S30). The CD spectrum of** 5** (Figure S31) was identical to that of** 1**, showing the 5*R*, 6*R*, and 7*R* absolute configuration. The stereochemistry of the C-14 secondary alcohol was deduced via the CD data of the in situ formed [Rh_2_(OCOCF_3_)_4_] complex with acetonide** 5a** (Figure 4). The Rh-complex of** 5a** displayed positive Cotton effects at near 322 nm, suggesting the 14*S* absolute configuration [17].

The structures of known compounds (**6**–**11**) were determined by NMR data analyses and comparison with the literature data [10–12].

*Compound* ***1***. Brown oil (MeOH); [*α*]_D_^25^  −43.1 (*c* 0.12, methanol); UV (methanol) *λ*_max_ (log⁡*ε*): 210 (3.83), 270 (3.20), 303 (3.02) nm; CD (*c* 5.5 × 10^−3^ M, methanol): *λ* (Δ*ε*) 220 (2.22), 240 (−0.44), 277 (0.78), 352 (0.22); IR (neat) *v*_max_: 3384 (br), 2937, 2878, 1680, 1447, 1382, 1263, 1022 cm^−1^; for ^1^H NMR and ^13^C NMR data see Table 1; Positive HR-ESI-MS: *m*/*z* 389.1587 [M + Na]^+^ (calcd. for C_19_H_26_O_7_Na, 389.1576).

*Compound* ***2***. Brown oil (MeOH); [*α*]_D_^25^ 5.0 (*c* 0.025, methanol); UV (methanol) *λ*_max_ (log⁡*ε*): 211 (3.03); CD (*c* 4.6 × 10^−3^ M, methanol): *λ* (Δ*ε*) 216 (0.20), 267 (6.85), 356 (0.21); IR (neat) *v*_max_: 3380 (br), 2963, 2933, 2878, 1689, 1436, 1381, 1253, 1081, 1026 cm^−1^; for ^1^H NMR and ^13^C NMR data see Table 1; Positive HR-ESI-MS: *m*/*z* 431.1684 [M + Na]^+^ (calcd. for C_21_H_28_O_8_Na, 431.1682).

*Compound* ***3***. Brown oil (MeOH); [*α*]_D_^25^  −140.0 (*c* 0.1, methanol); UV (methanol) *λ*_max_ (log⁡*ε*): 211 (4.80) nm; CD (*c *2.3 × 10^−3^ M, methanol): *λ* (Δ*ε*) 207 (3.43), 239 (−13.99); IR (neat) *v*_max_: 3383 (br), 2965, 2933, 2660, 1694, 1652, 1557, 1377, 1252, 1130, 1025 cm^−1^; for ^1^H NMR and ^13^C NMR data see Table 1; Positive HR-ESI-MS: *m*/*z* 433.1848 [M + Na]^+^ (calcd. for C_21_H_30_O_8_Na, 433.1838).

*Compound* ***4***. Brown oil (MeOH); [*α*]_D_^25^ 250.0 (*c* 0.1, methanol); UV (methanol) *λ*_max_ (log⁡*ε*): 211 (4.54) nm; CD (*c* 2.3 × 10^−3^ M, methanol): *λ* (Δ*ε*) 223 (2.50), 268 (8.45), 354 (1.15); IR (neat) *v*_max_: 3353 (br), 2965, 2934, 1684, 1646, 1447, 1374, 1256, 1130, 1079, 1026 cm^−1^; for ^1^H NMR and ^13^C NMR data see Table 2; Positive HR-ESI-MS: *m*/*z* 431.1692 [M + Na]^+^ (calcd. for C_21_H_28_O_8_Na, 431.1682).

*Compound* ***5***. Brown oil (MeOH); [*α*]_D_^25^  −80.0 (*c* 0.1, methanol); UV (methanol) *λ*_max_ (log⁡*ε*): 213 (4.22), 299 (3.56) nm; CD (*c* 5.1 × 10^−3^ M, methanol): *λ* (Δ*ε*) 223 (1.33), 273 (1.23); IR (neat) *v*_max_: 3393 (br), 2959, 2933, 2873, 1680, 1647, 1437, 1380, 1260, 1022 cm^−1^; for ^1^H NMR and ^13^C NMR data see Table 2; Positive HR-ESI-MS: *m*/*z* 389.1584 [M + Na]^+^ (calcd. for C_19_H_26_O_7_Na, 389.1576).

### 3.2. Proposed Biosynthetic Pathway of** 1**–**11**

**Figure 5:**
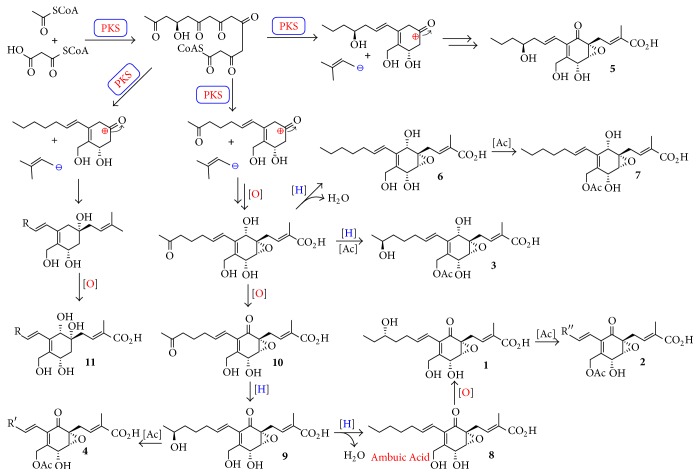
The possible biosynthetic pathway of ambuic acid and its analogs.

Analysis of the structural features of ambuic acid and its analogs implied their biosynthesis originated from polyketide-terpene hybrid, and the biogenetic pathway was first suggested as shown in Figure 5. Ambuic acid and its analogs possess a highly functionalized cyclohexenone ring, and, considering their biosynthesis, their biosynthesis demonstrated that the postmodification enzymes especially oxidase play a vital role in the formation of chemical diversity.

### 3.3. Biological Activities

Compounds** 1**–**11** were evaluated against several tumor cells including A549, HepG 2, and Hela cell lines by MTT colorimetric method [20] using etoposide as positive control (IC_50_ values 16.46 ± 1.22, 16.11 ± 0.10, and 15.00 ± 0.23*μ*M, respectively). Unfortunately, none of these ambuic acid analogs showed significant cytotoxicity (IC_50_ > 40 *μ*M) against the tested cancer cell lines. Compounds** 1**–**11** were also evaluated against* Staphylococcus aureus* (DSM 799),* Escherichia coli* (DSM 1116), and* Bacillus subtilis* (DSM 1088) without any bioactivities (MIC > 64 *µ*g/mL, positive control as streptomycin, MIC values 5, 1, and 5 *µ*g/mL, resp.). In addition, the antifungal activities against six plant pathogens* Botrytis cinerea*,* Verticillium dahlia*,* Fusarium oxysporum*,* Alternaria solani*,* Fusarium gramineum*, and* Rhizoctonia solani* displayed that** 1** and** 5** exhibited pronounced biological effects against* F. oxysporum* with MIC value of 8 *µ*g/mL, whereas** 5** and** 6** can potently inhibit* F. gramineum* at concentration of 8 *µ*g/mL, compared with the positive control ketoconazole (MIC value of 8 *µ*g/mL) (Table 3). Our biological evaluation results further supported that ambuic acid analogs might possess potential bioactivities against pathogens, without biological effects against bacteria.

## 4. Conclusion

In summary, five new metabolites (**1**–**5**) with highly functionalized groups, together with six known derivatives (**6**–**11**), were isolated from the endolichenic fungus* Pestalotiopsis* sp. Cytotoxicity and antimicrobial bioassays were performed and the results revealed that compound** 1** and** 5** can significantly inhibit the growth of plant pathogenic fungus* F. oxysporum*, and compounds** 5** and** 6** potently inhibited* F. gramineum*, with MIC value of 8 *µ*g/mL, and these results might reveal the possible ecological role of endolichenic fungus for providing chemical protection for its host plants in the fungus-plant relationship. Our results also implied the potentiality of compound** 1**,** 5,** and** 6** as suitable fungicides in application of agricultural crop protection. Analysis of the structural features of ambuic acid and its analogs implied their biosynthesis originated from polyketide-terpene hybrid, and the biogenetic pathway was suggested for the first time, which is helpful in its further biosynthesis research, because ambuic acid derivatives were unique to the genus* Pestalotiopsis*, which suggest their contributions as diagnostic metabolites in the chemical taxonomy of* Pestalotiopsis* sp.

## Supplementary Material

NMR and CD data of new compounds **1**–**5**, ^1^HNMR spectral of **1a** and **5a**.

## Figures and Tables

**Table 1:** ^1^H and ^13^C NMR spectroscopic data of compounds **1**–**3** in CD_3_OD^a^.

Number	**1**		**2**		**3**	
	*δ*_C_	*δ*_H_ (mult., *J* in Hz)	*δ*_C_	*δ*_H_ (mult., *J* in Hz)	*δ*_C_	*δ*_H_ (mult., *J* in Hz)
(1)	171.2		172.8		171.9	
(2)	131.9		133.3		132.4	
(3)	136.5	6.69, t (7.8)	134.3	6.61, t (7.5)	136.7	6.82, t (7.5)
(4)	28.8	2.78, dd (15.9, 7.6)	28.6	2.75, dd (15.9, 8.0)	31.8	2.61, dd (16.0, 8.0)
		2.79, dd (15.9, 7.6)		2.80, dd (15.9, 8.0)		3.00, dd (16.0, 8.0)
(5)	61.2		61.5		62.2	
(6)	61.1	3.76, d (3.0)	61.0	3.75, d (3.0)	60.7	3.35, br s
(7)	65.9	4.83, br s	65.8	4.72, br s	67.2	4.63, br s
(8)	150.8		145.5		128.5	
(9)	131.9		134.9		136.6	
(10)	196.0		195.8		67.0	4.49, s
(11)	122.9	6.17, d (16.0)	123.0	6.17 d (16.0)	127.2	6.34, d (15.5)
(12)	139.9	5.88, dt (16.0, 8.4)	140.5	5.83, dt (16.0, 7.0)	135.5	6.01, dt (15.0, 8.0)
(13)	30.8	2.23, m	30.8	2.17, m	34.5	2.19, m
(14)	37.3	1.57, m	37.3	1.56, m	26.5	1.47, m
(15)	73.2	3.48, m	73.2	3.46, m	39.6	1.46, m
(16)	31.1	1.47, m	31.1	1.47, m	68.4	3.73, m
(17)	10.4	0.94, t (8.4)	10.4	0.94, t (8.4)	23.6	1.14, d (6.0)
(18)	60.3	4.41, d (13.0)	62.5	4.92, d (12.5)	60.8	4.86, s
		4.52, d (13.0)		4.95, d (12.5)		
(19)	12.8	1.86, s	13.1	1.86, s	13.0	1.86, s
(20)			172.4		172.5	
(21)			20.7	2.06, s	20.9	2.02, s

^a^ ^1^H-NMR was recorded at 500 MHz; ^13^C-NMR was recorded at 125 MHz.

**Table 2:** ^1^H and ^13^C NMR spectroscopic data of compounds **4** and **5** in CD_3_OD^a^.

Number	**4**		**5**	
	*δ*_C_	*δ*_H_ (mult., *J* in Hz)	*δ*_C_	*δ*_H_ (mult., *J* in Hz)
(1)	171.6		171.6	
(2)	132.4		132.2	
(3)	136.0	6.66, t (7.0)	136.2	6.67, t (7.0)
(4)	28.6	2.77, dd (15.5, 7.0)	28.8	2.75, dd (15.5, 7.5)
		2.80, dd (15.5, 7.0)		2.82, dd (15.5, 7.5)
(5)	61.4		61.3	
(6)	61.0	3.75, d (3.0)	61.1	3.76, d, (3.0)
(7)	65.8	4.72, br s	65.9	4.83, br s
(8)	145.5		151.1	
(9)	134.3		131.8	
(10)	195.4		196.1	
(11)	123.0	6.32, d (16.5)	124.9	6.19, d (16.0)
(12)	140.6	5.86, dt (16.0, 7.0)	136.5	5.91, dt (16.0, 7.5)
(13)	34.5	2.18, m	42.6	2.30, m
(14)	26.4	1.47, m	71.9	3.64, m
(15)	39.6	1.45, m	40.2	1.44, m
(16)	68.4	3.74, m	19.9	1.48, m
(17)	23.6	1.14, t (6.5)	14.4	0.93, t (7.0)
(18)	62.5	4.91, d (12.5)	60.3	4.41, d (12.5)
		4.96, d (12.5)		4.53, d (12.5)
(19)	12.9	1.86, s	12.8	1.86, s
(20)	172.4			
(21)	20.7	2.05, s		

^a^ ^1^H-NMR was recorded at 500 MHz; ^13^C-NMR was recorded at 125 MHz.

**Table 3:** Minimum Inhibitory Concentrations (MIC) of compounds **1**–**11** against six plant pathogenic fungi^a^.

Compounds	*Botrytis cinerea*	*Verticillium dahlia*	*Fusarium oxysporum*	*Alternaria solani*	*Fusarium gramineum*	*Rhizoctonia solani*
**1**	>64	64	8	>64	>64	>64
**2**	>64	64	32	>64	>64	>64
**3**	>64	64	64	>64	>64	64
**4**	>64	32	64	>64	>64	>64
**5**	>64	16	8	>64	8	64
**6**	>64	64	64	>64	8	64
**7**	>64	>64	>64	>64	64	32
**8**	>64	>64	64	>64	64	32
**9**	>64	64	16	>64	32	>64
**10**	>64	64	>64	>64	64	>64
**11**	>64	64	>64	>64	32	64
Ketoconazole	16	1	8	16	8	8

^a^All values are in *μ*g/mL.
