# Supplementary material for: Polyketide-Terpene Hybrid Metabolites from an Endolichenic Fungus Pestalotiopsis sp
Source: Biomed Res Int. 2017 May 16;2017:6961928. doi: 10.1155/2017/6961928 (PMC5448061; doi:10.1155/2017/6961928)

Supplementary Information

Polyketide-terpene Hybrid Metabolites From an Endolichenic Fungus *Pestalotiopsis* sp.

Chao Yuan[1, #], Gang Ding[2, #], Hai-Ying Wang[3], Yu-Hua Guo[2], Hai Shang[2], Xiao-Jun Ma[2, *], and Zhong-Mei Zou[2, *]

[1] Institute of Medicinal Plant Development Yunnan Branch, Chinese Academy of Medical Sciences and Peking Union Medical College, Jinghong 666100, China

[2] Institute of Medicinal Plant Development, Chinese Academy of Medical Sciences and Peking Union Medical College, Beijing 100193, China

[3] College of Life Sciences, Shandong Normal University, No. 88 East Wenhua Road, Jinan 250014, China

[#] Authors contributing equally to this work.
* Authors to whom correspondence should be addressed;
Correspondence and requests for materials should be addressed to Zhong-Mei Zou (E-mail: zmzou@implad.ac.cn) or Xiao-Jun Ma (E-mail: mayixuan10@163.com).

Contents

Figure S1 ^{1}H NMR spectrum of compound **1** (500 MHz, CD_3OD)

Figure S2 ^{13}C NMR spectrum of compound **1** (500 MHz, CD_3OD)

Figure S3 ^{1}H-^{1}HCOSY spectrum of compound **1** (500 MHz, CD_3OD)

Figure S4 HMBC spectrum of compound **1** (500 MHz, CD_3OD)

Figure S5 HSQC spectrum of compound **1** (500 MHz, CD_3OD)

Figure S6 ROESY spectrum of compound **1** (500 MHz, CD_3OD)

Figure S7 CD spectrum of compound **1** in methanol

Figure S8 ^{1}H NMR spectrum of compound **2** (500 MHz, CD_3OD)

Figure S9 ^{13}C NMR spectrum of compound **2** (500 MHz, CD_3OD)

Figure S10 HMBC spectrum of compound **2** (500 MHz, CD_3OD)

Figure S11 HSQC spectrum of compound **2** (500 MHz, CD_3OD)

Figure S12 ROESY spectrum of compound **2** (500 MHz, CD_3OD)

Figure S13 CD spectrum of compound **2** in methanol

Figure S14 ^{1}H NMR spectrum of compound **3** (500 MHz, CD_3OD)

Figure S15 ^{13}C NMR spectrum of compound **3** (500 MHz, CD_3OD)

Figure S16 HMBC spectrum of compound **3** (500 MHz, CD_3OD)

Figure S17 HSQC spectrum of compound **3** (500 MHz, CD_3OD)

Figure S18 ROESY spectrum of compound **3** (500 MHz, CD_3OD)

Figure S19 CD spectrum of compound **3** in methanol

Figure S20 ^{1}H NMR spectrum of compound **4** (500 MHz, CD_3OD)

Figure S21 ^{13}C NMR spectrum of compound **4** (500 MHz, CD_3OD)

Figure S22 HMBC spectrum of compound **4** (500 MHz, CD_3OD)

Figure S23 HSQC spectrum of compound **4** (500 MHz, CD_3OD)

Figure S24 ROESY spectrum of compound **4** (500 MHz, CD_3OD)

Figure S25 CD spectrum of compound **4** in methanol

Figure S26 ^{1}H NMR spectrum of compound **5** (500 MHz, CD_3OD)

Figure S27 ^{13}C NMR spectrum of compound **5** (500 MHz, CD_3OD)

Figure S28 HMBC spectrum of compound **5** (500 MHz, CD_3OD)

Figure S29 HSQC spectrum of compound **5** (500 MHz, CD_3OD)

Figure S30 ROESY spectrum of compound **5** (500 MHz, CD_3OD)

Figure S31 CD spectrum of compound **5** in methanol

Figure S32 ^{1}H NMR spectrum of compound **1a** (500 MHz, CD_3OD)

Figure S33 ^{1}H NMR spectrum of compound **5a** (500 MHz, CD_3OD)

Figure S1

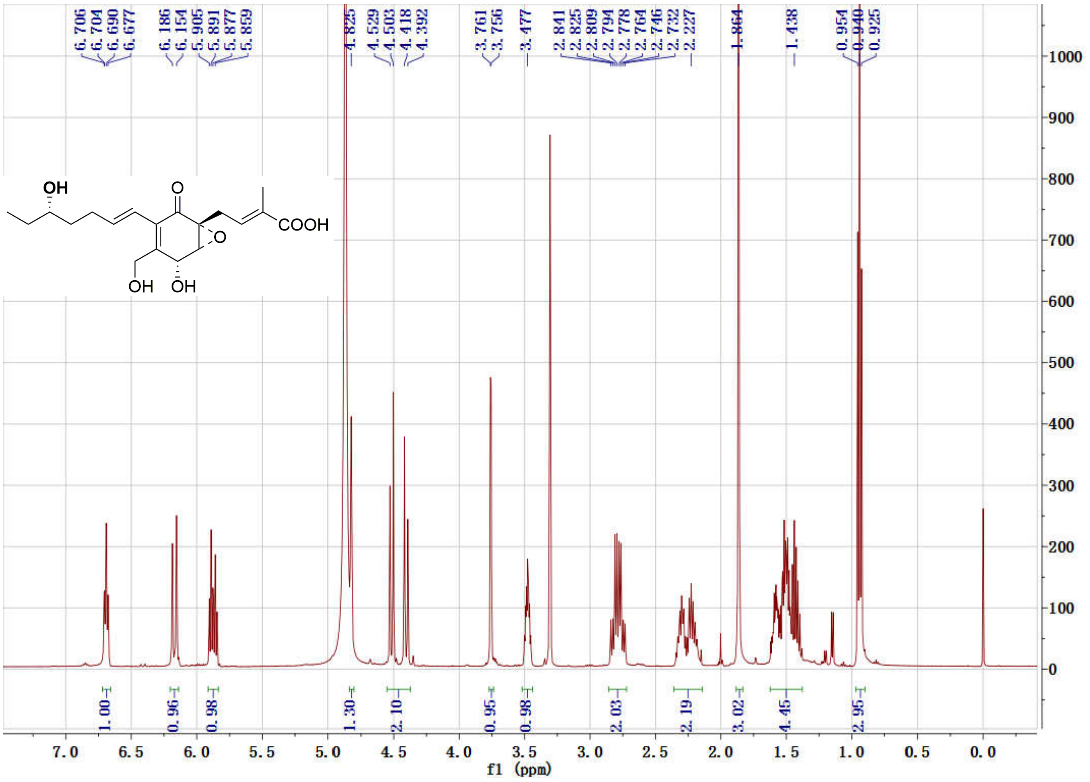

Figure S2

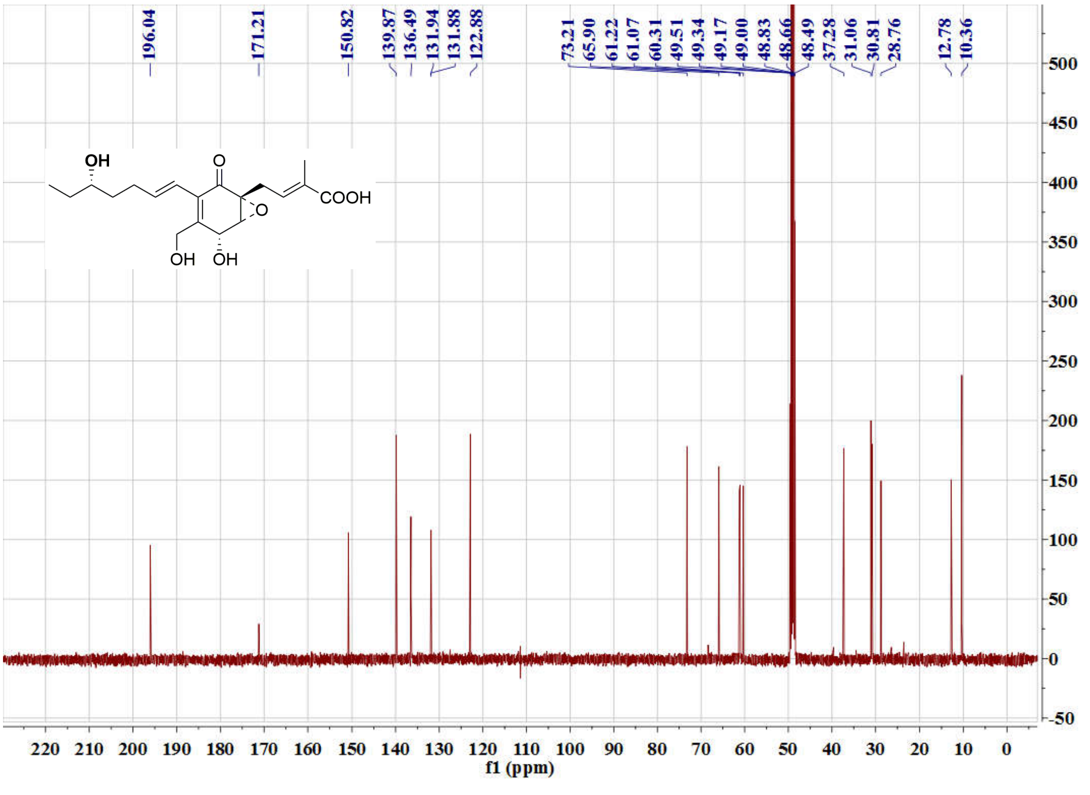

Figure S3

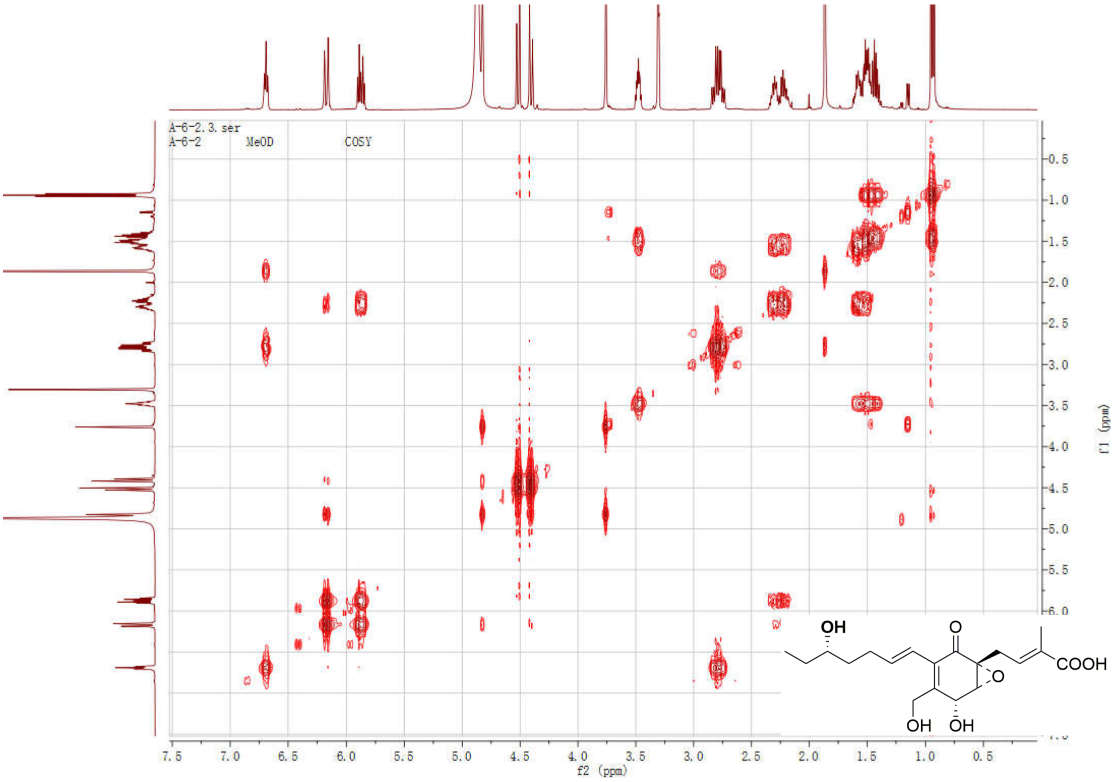

Figure S4

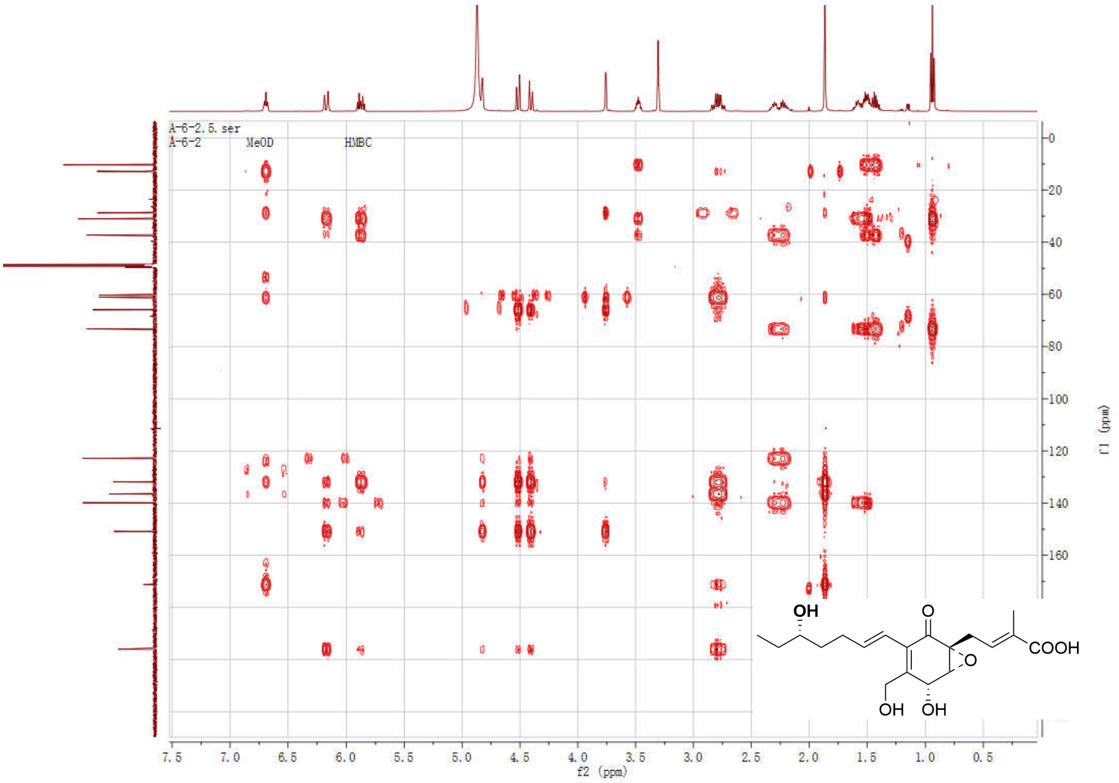

Figure S5

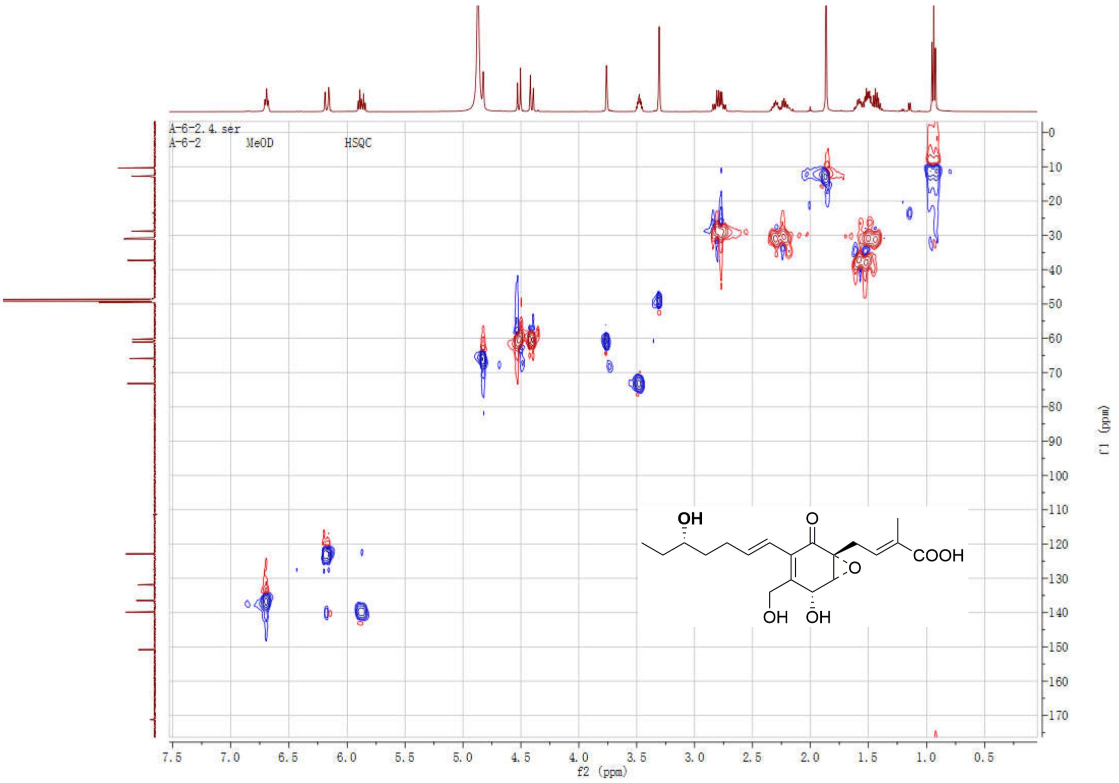

Figure S6

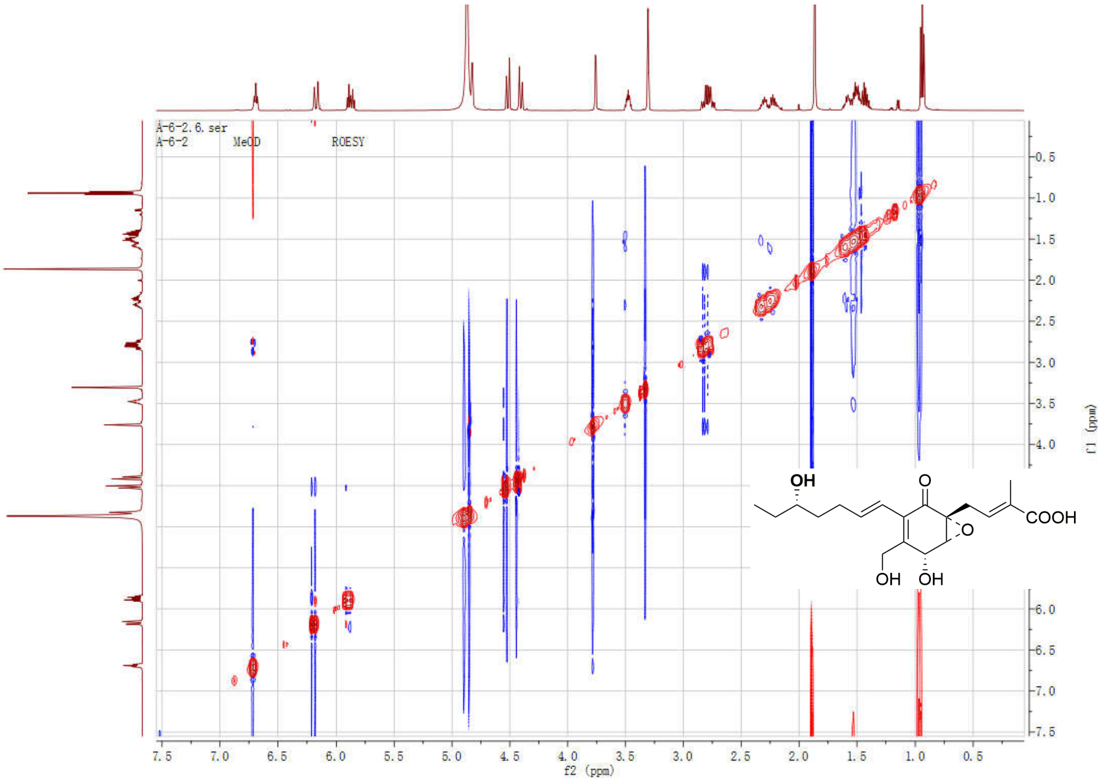

Figure S7

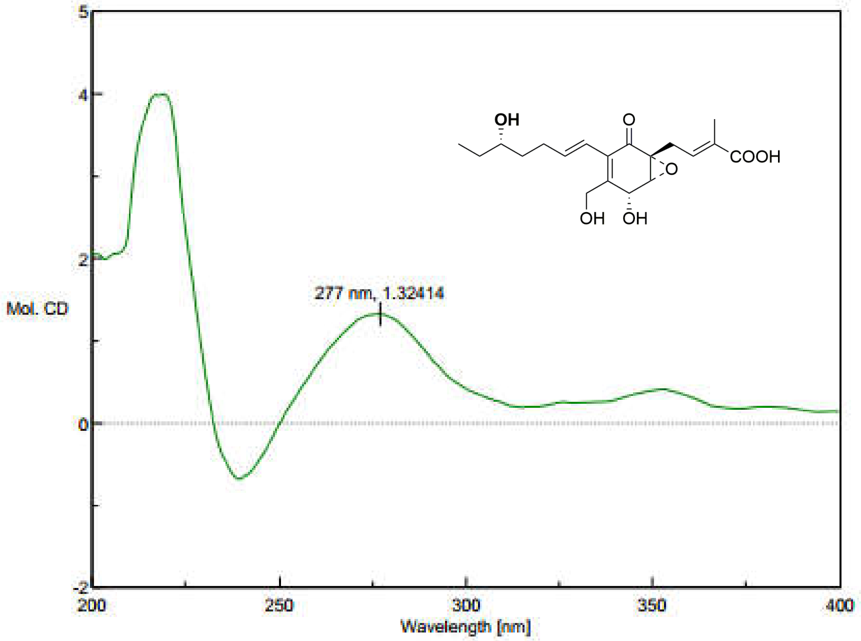

Figure S8

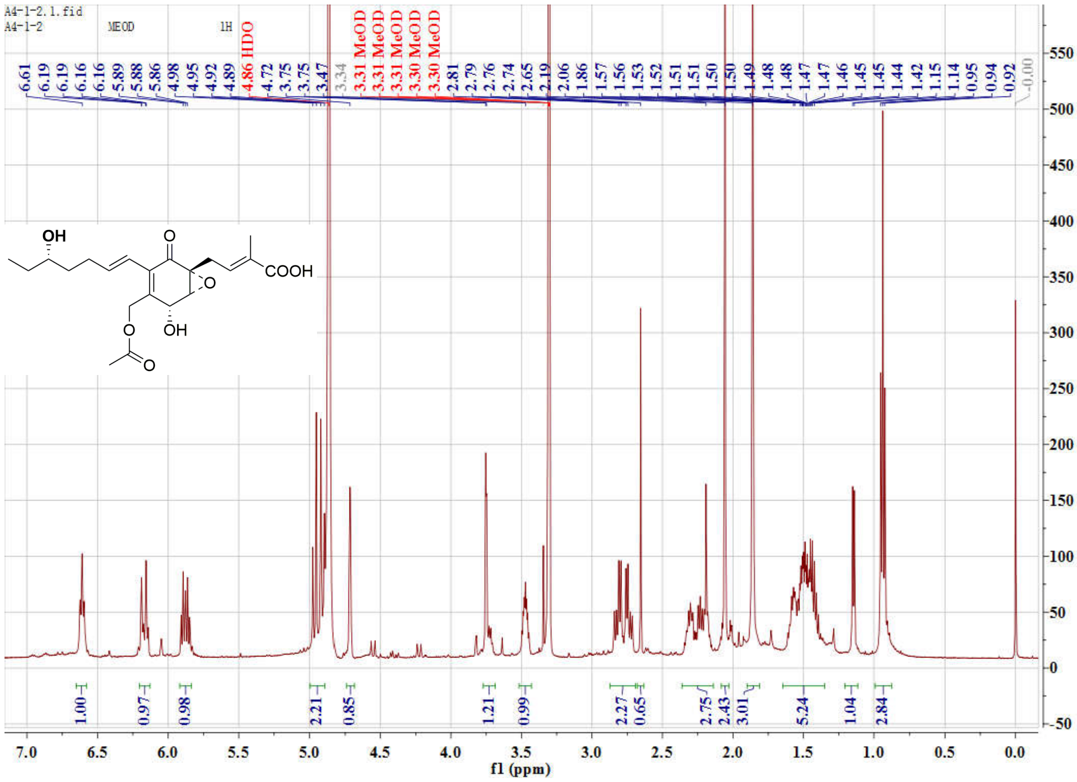

Figure S9

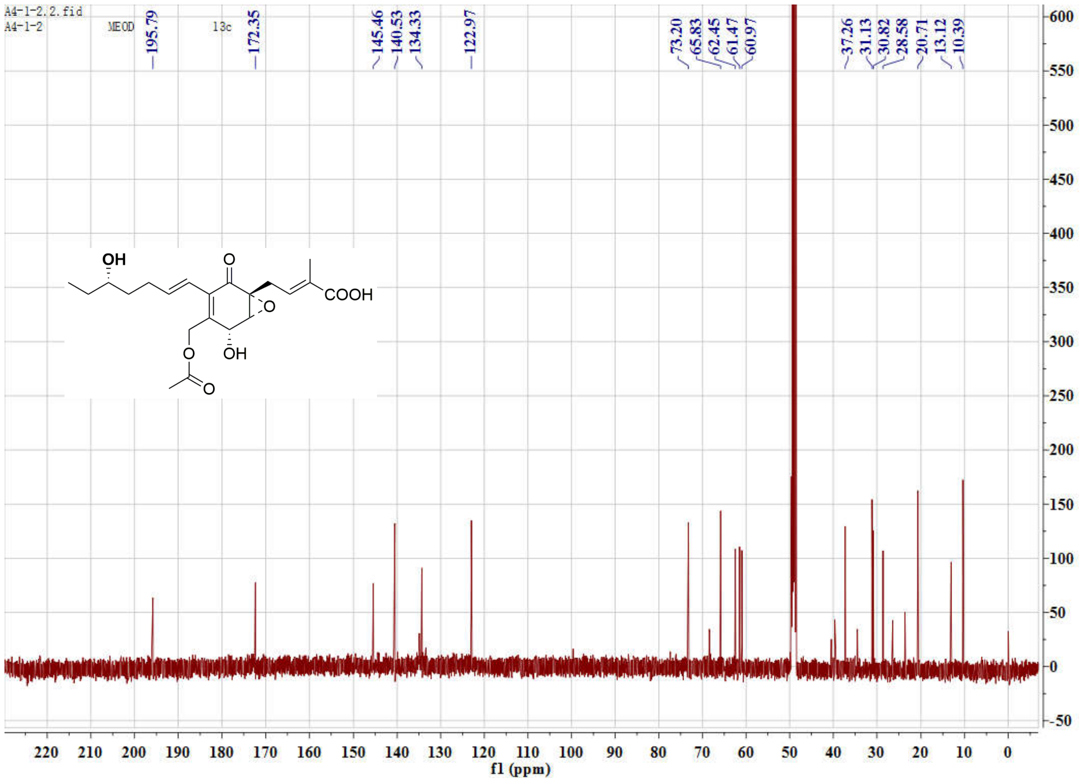

Figure S10

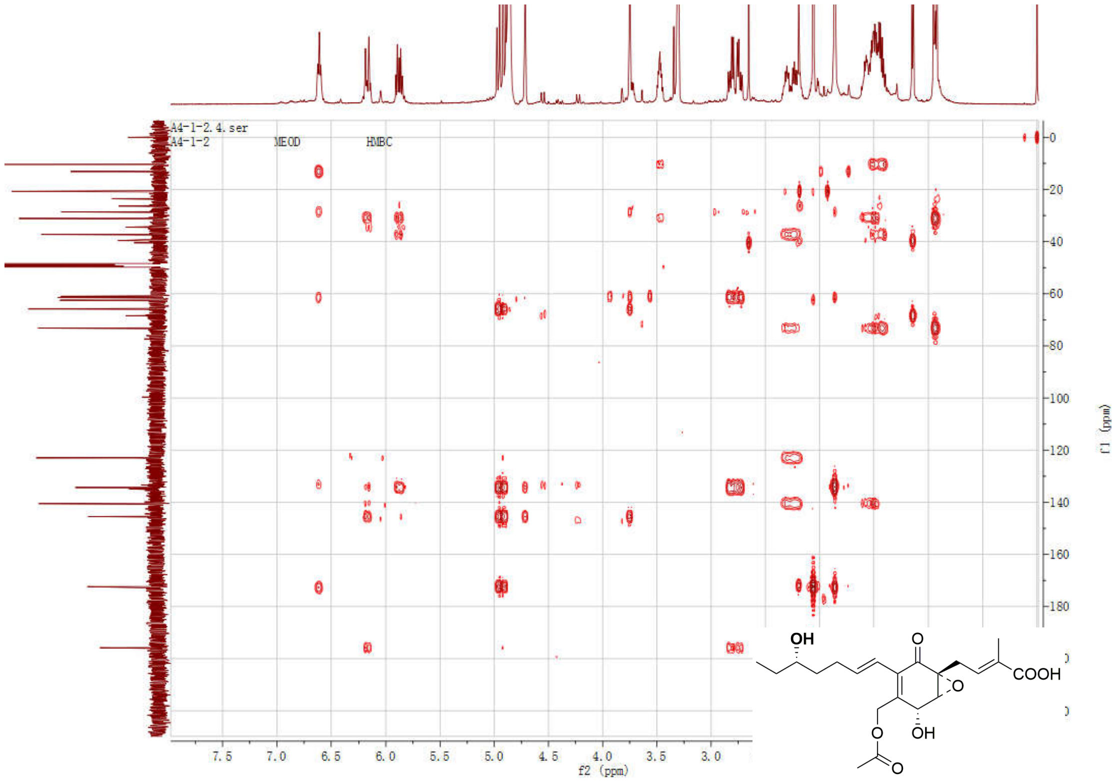

Figure S11

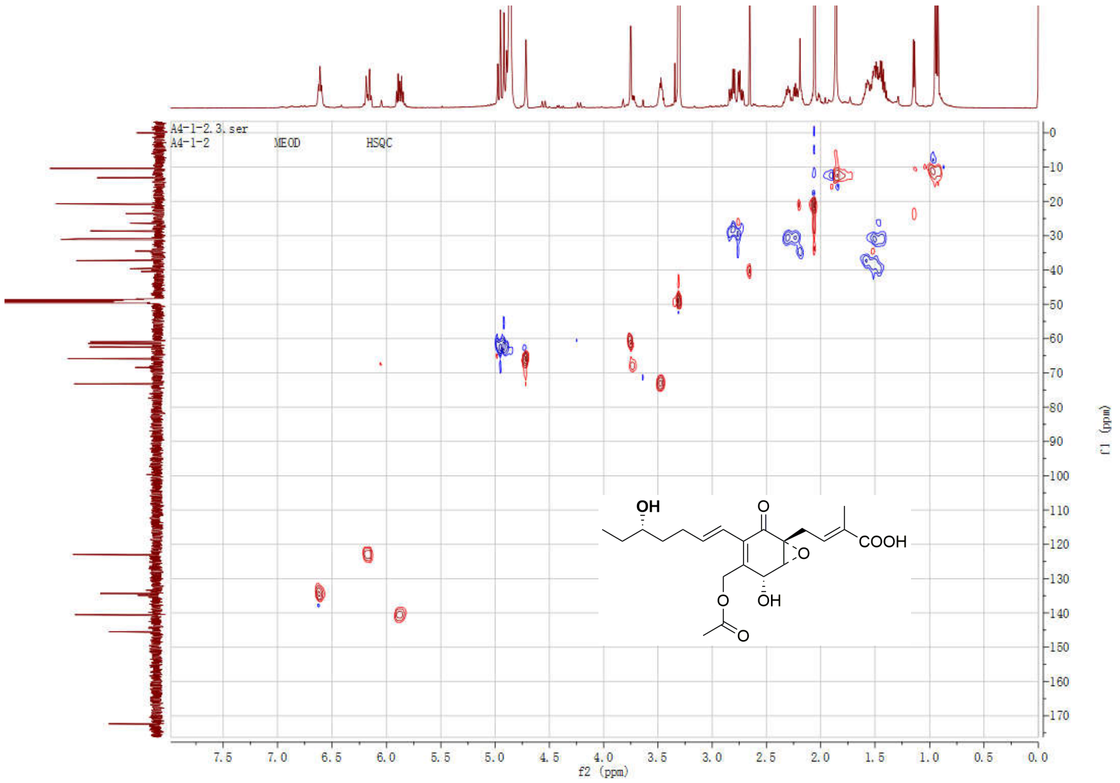

Figure S12

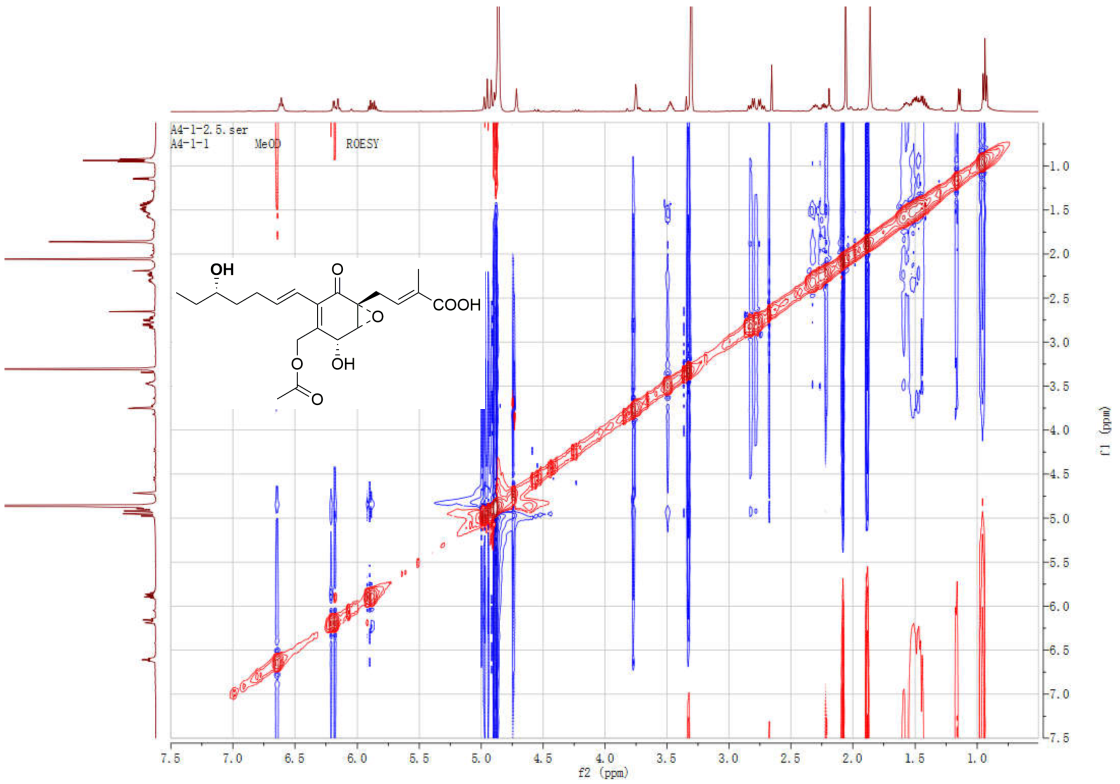

Figure S13

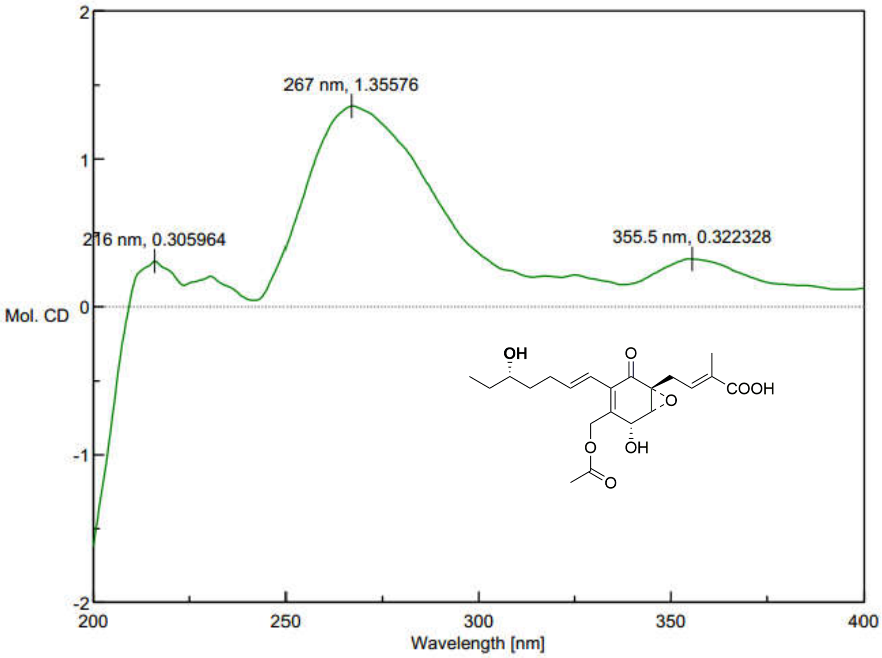

Figure S14

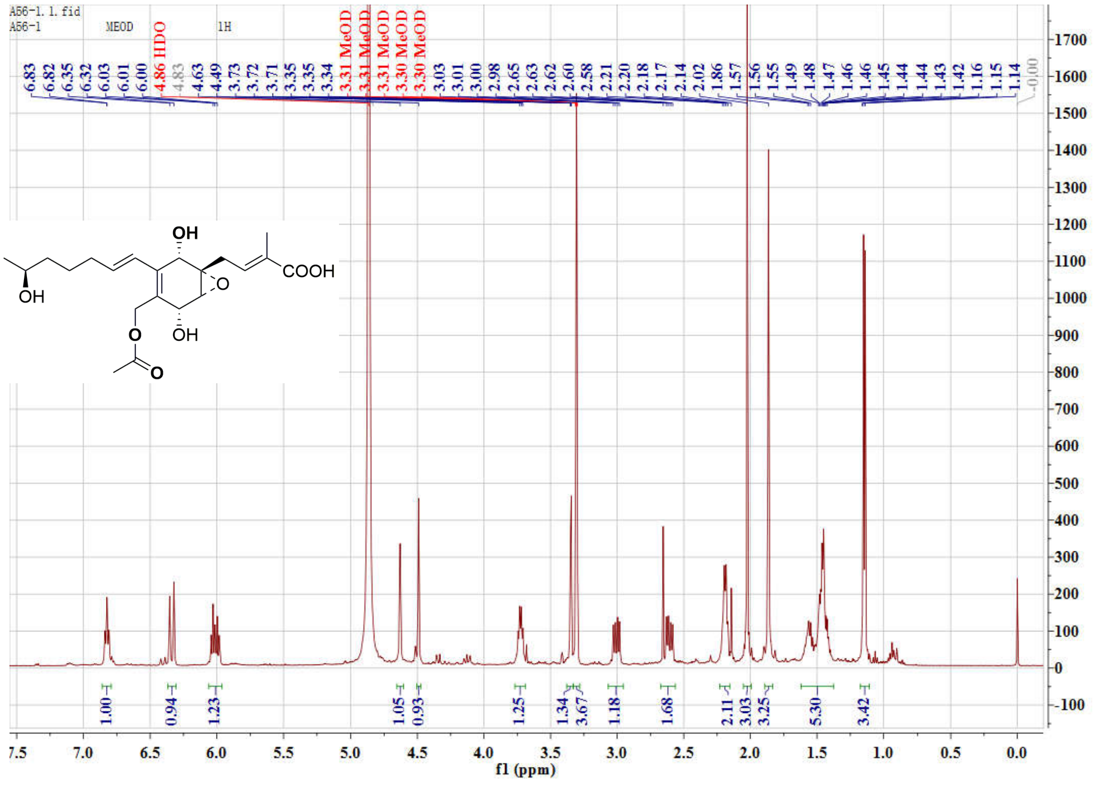

Figure S15

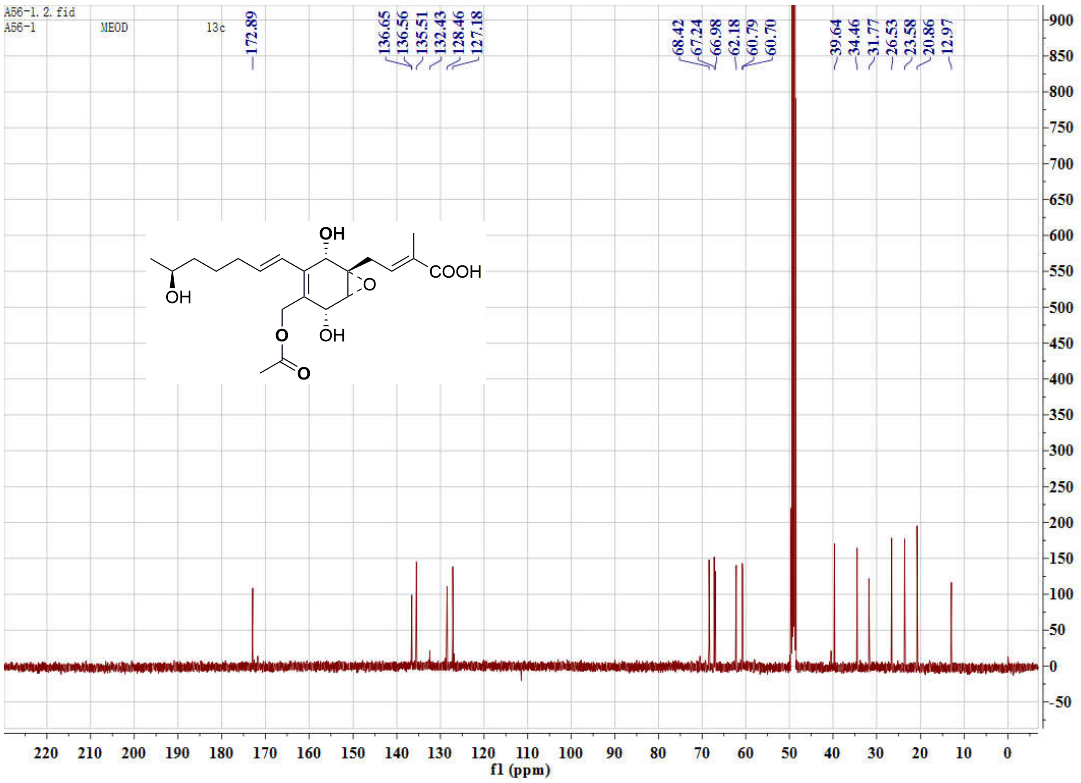

Figure S16

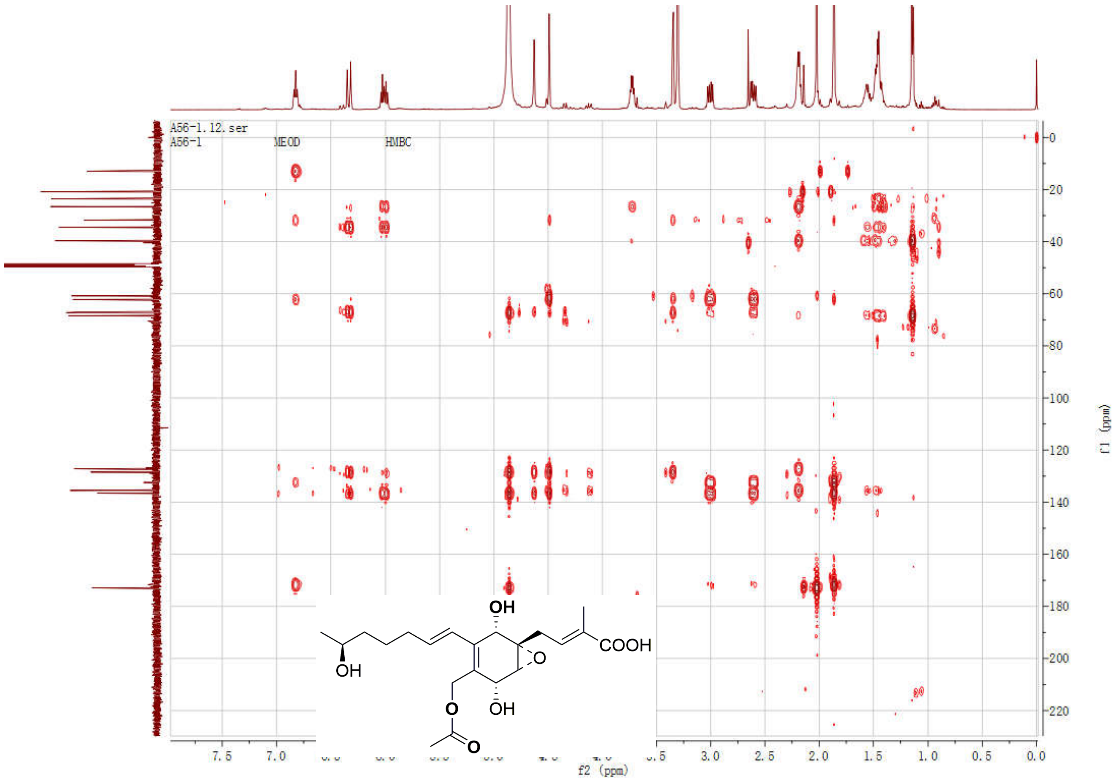

Figure S17

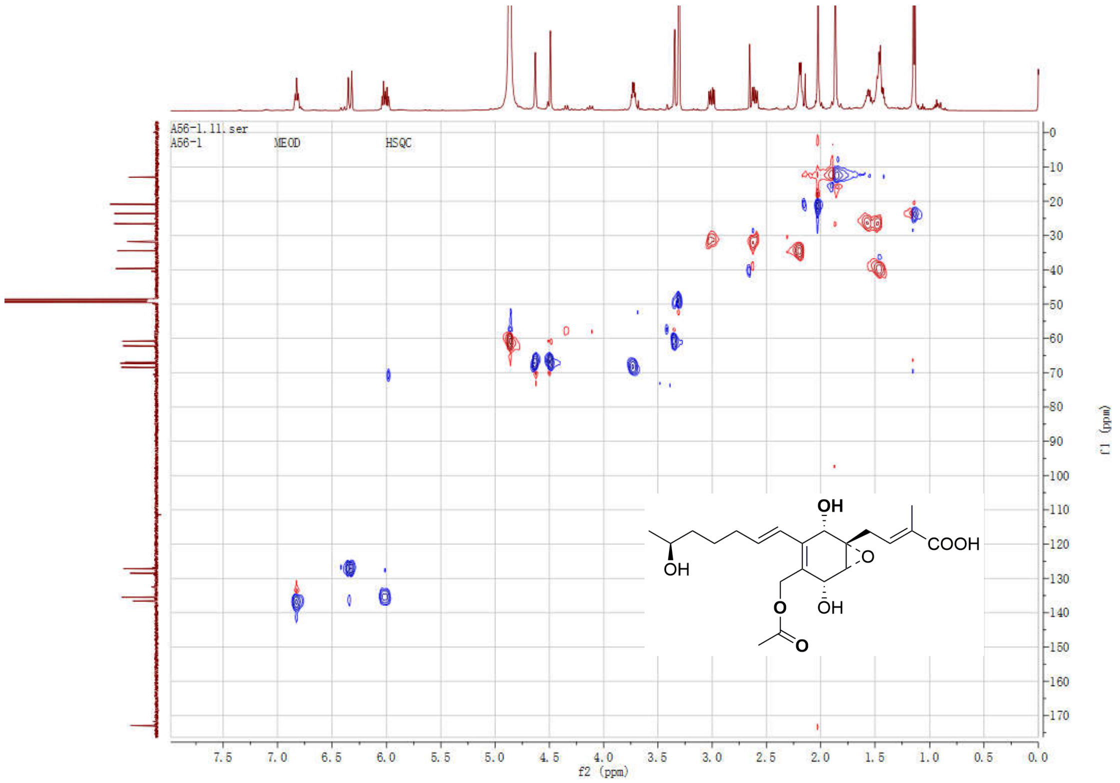

Figure S18

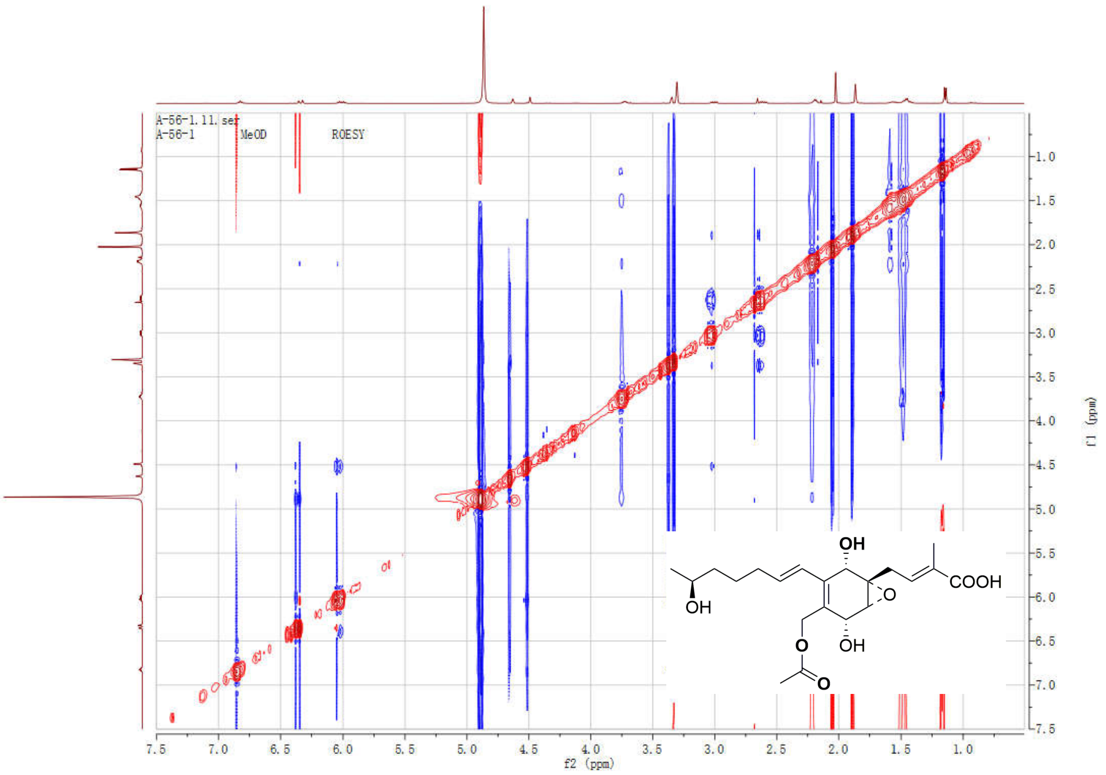

Figure S19

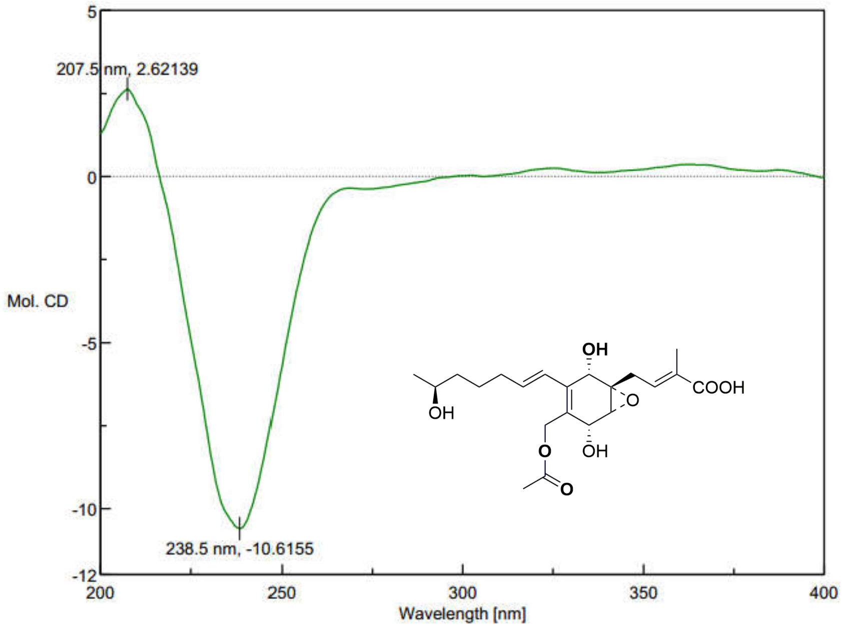

Figure S20

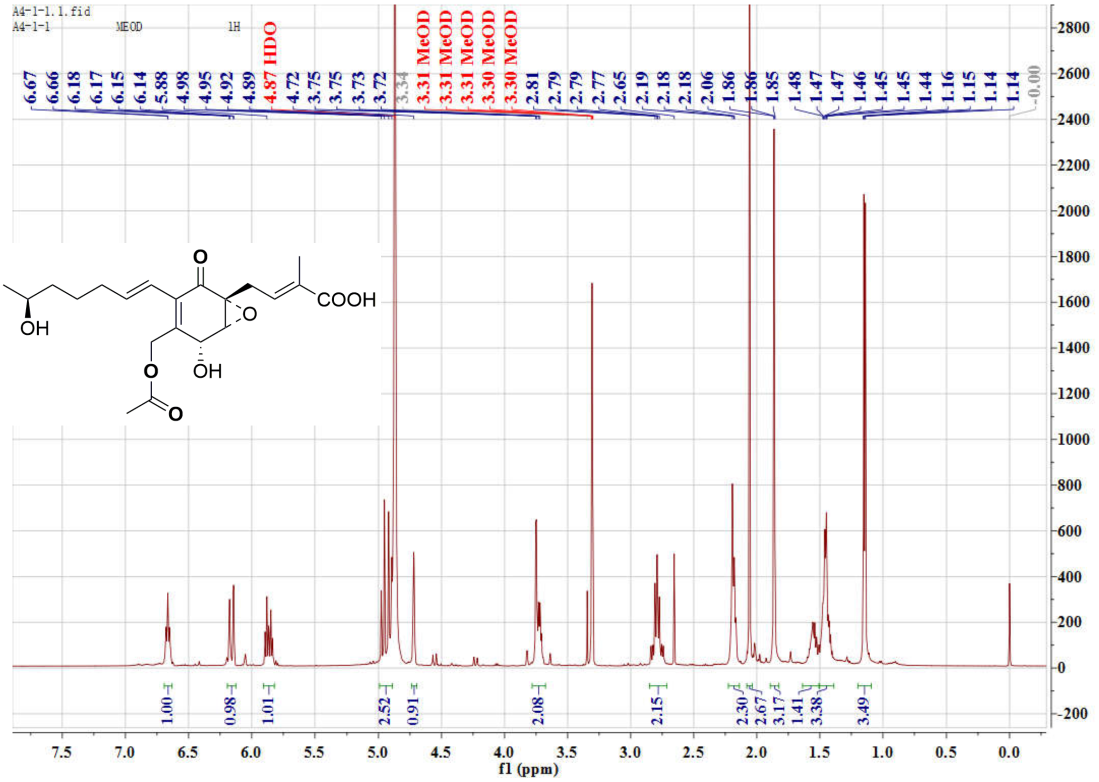

Figure S21

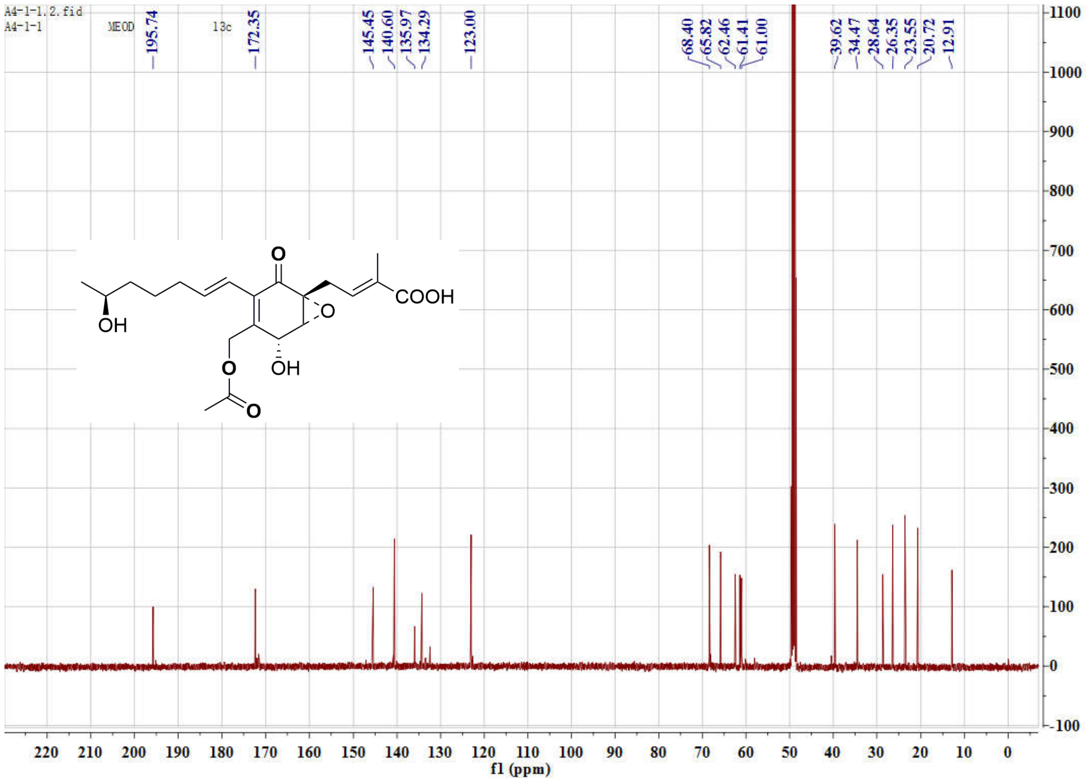

Figure S22

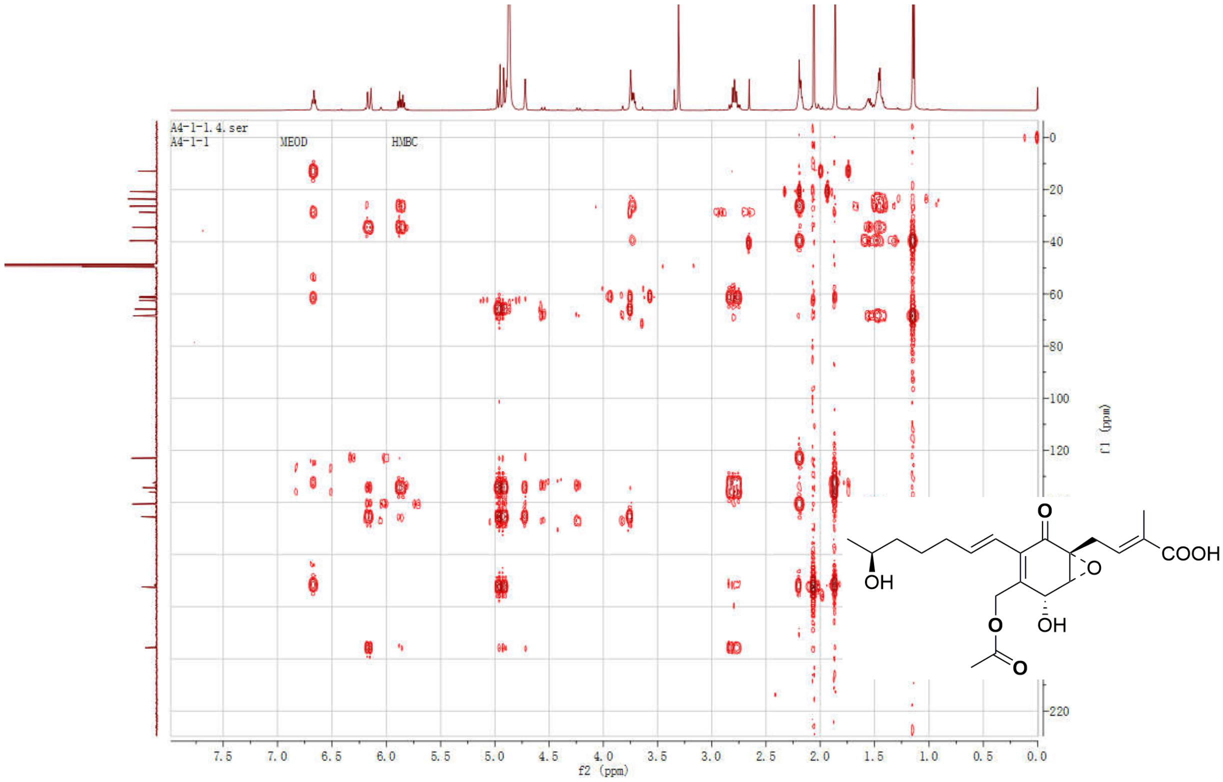

Figure S23

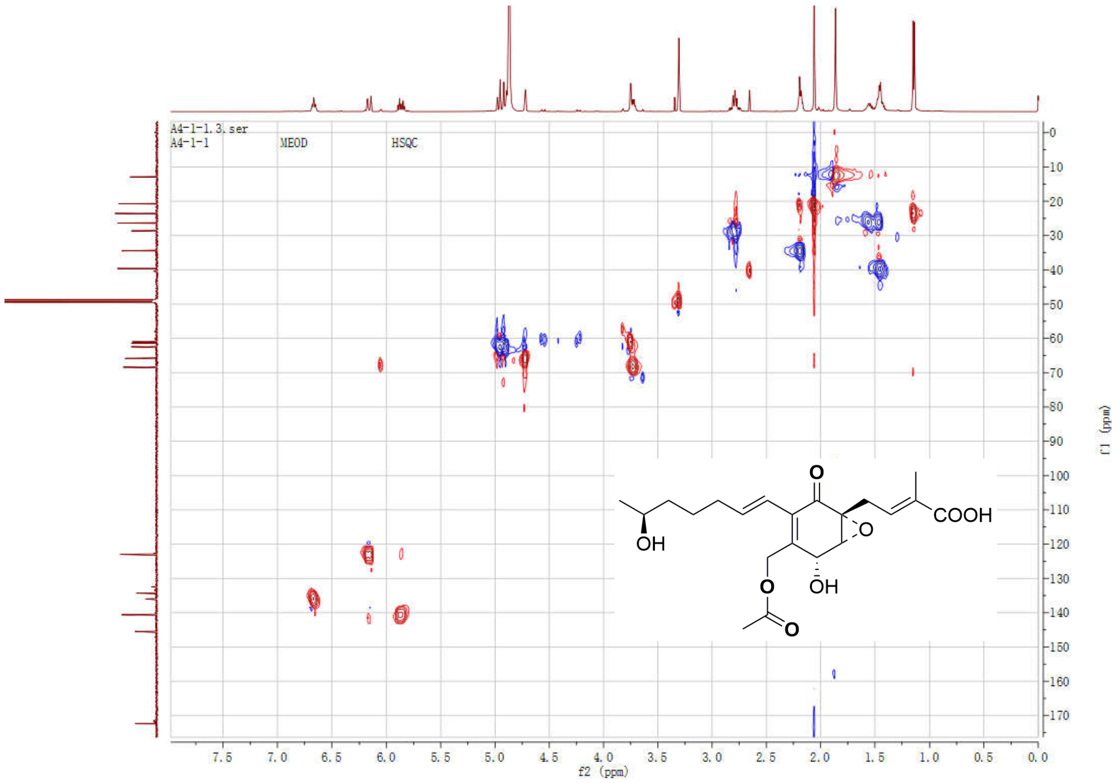

Figure S24

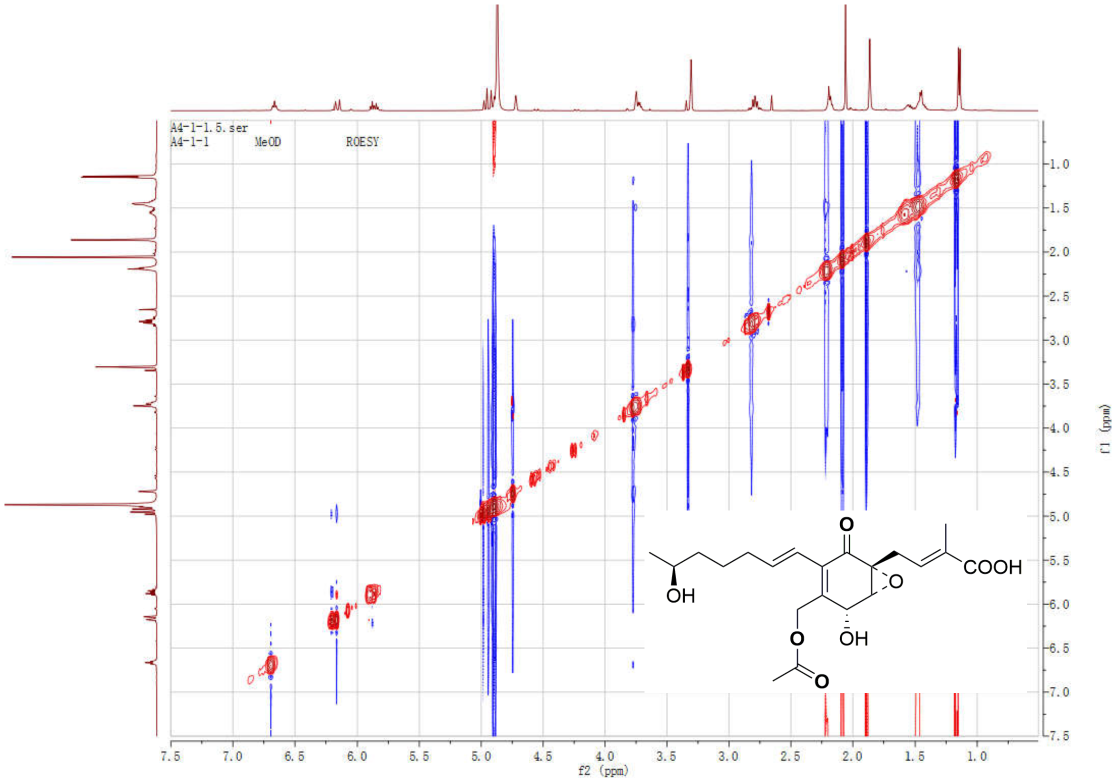

Figure S25

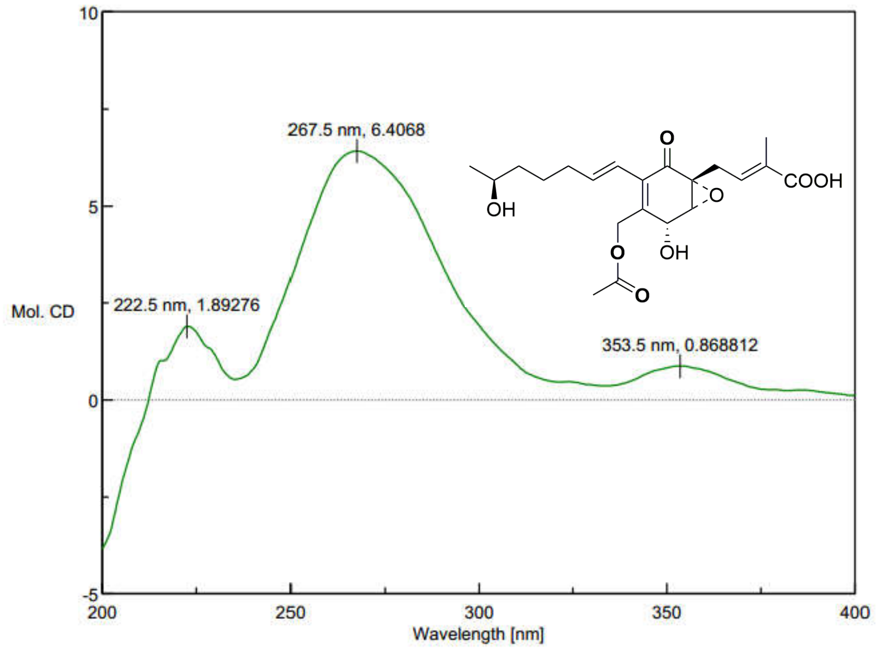

Figure S26

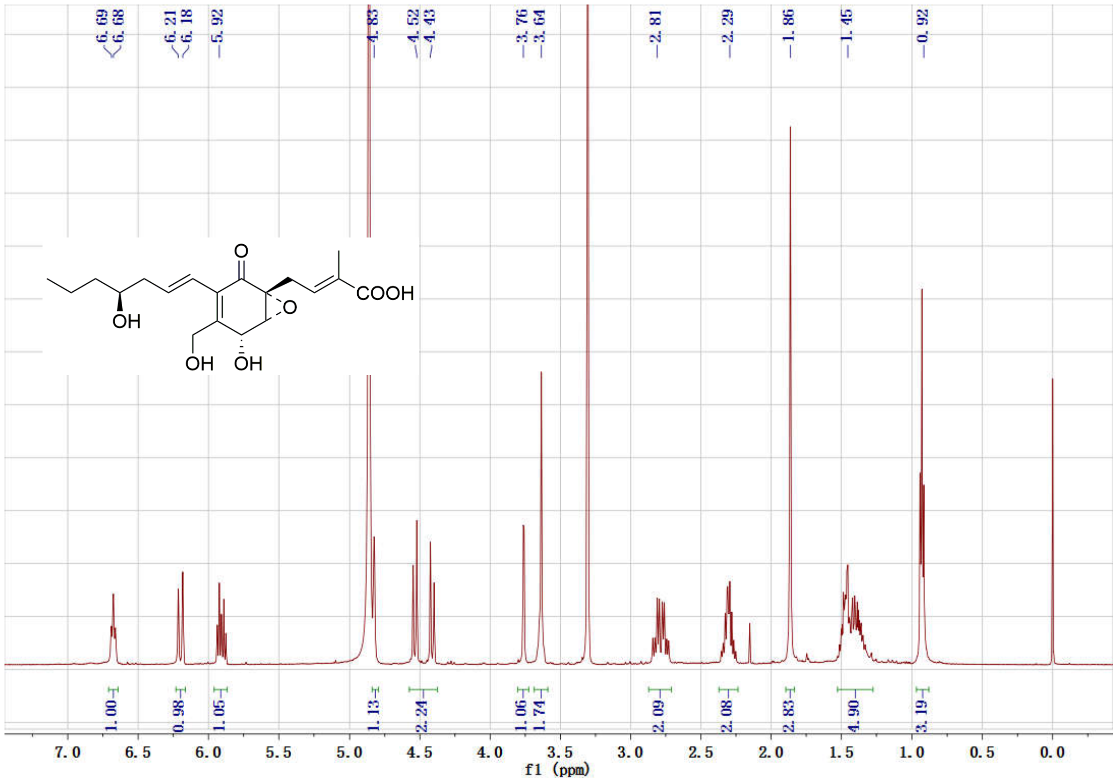

Figure S27

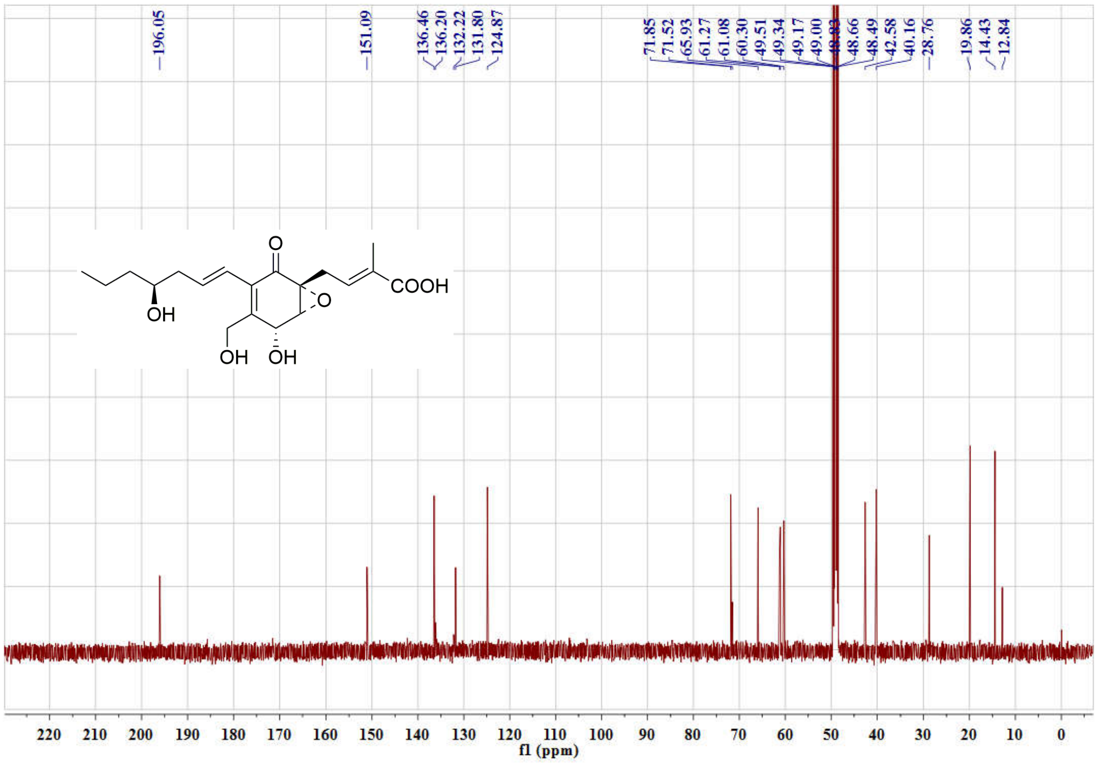

Figure S28

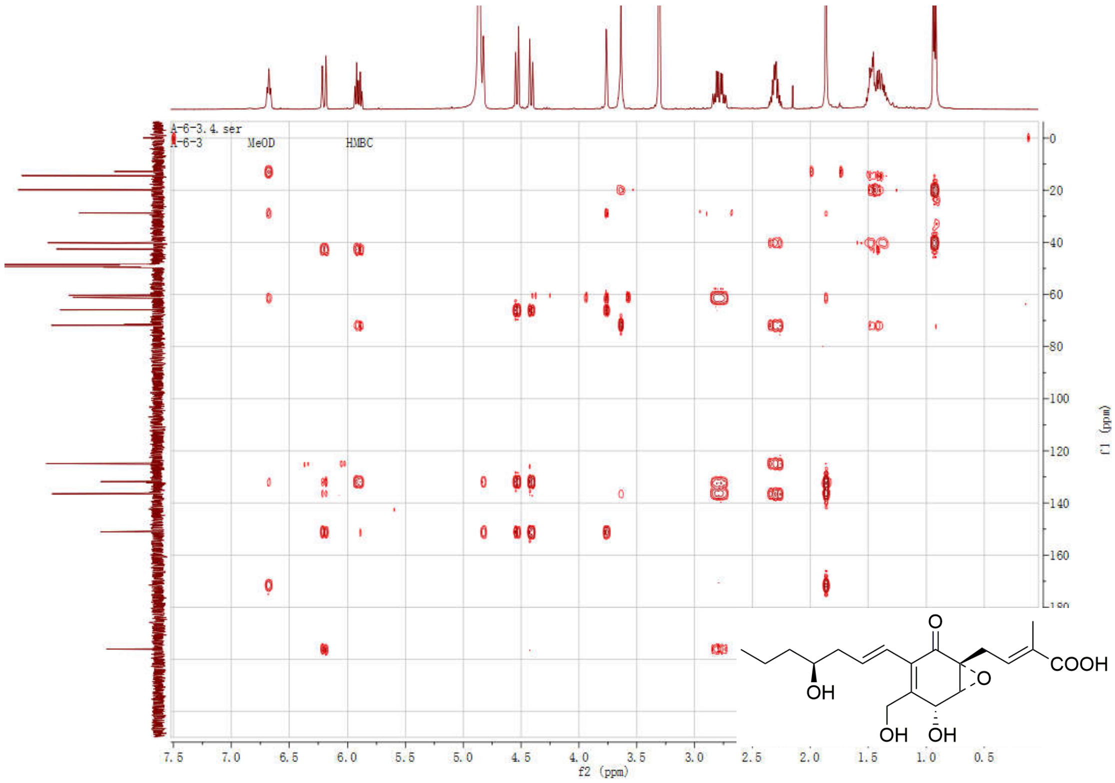

Figure S29

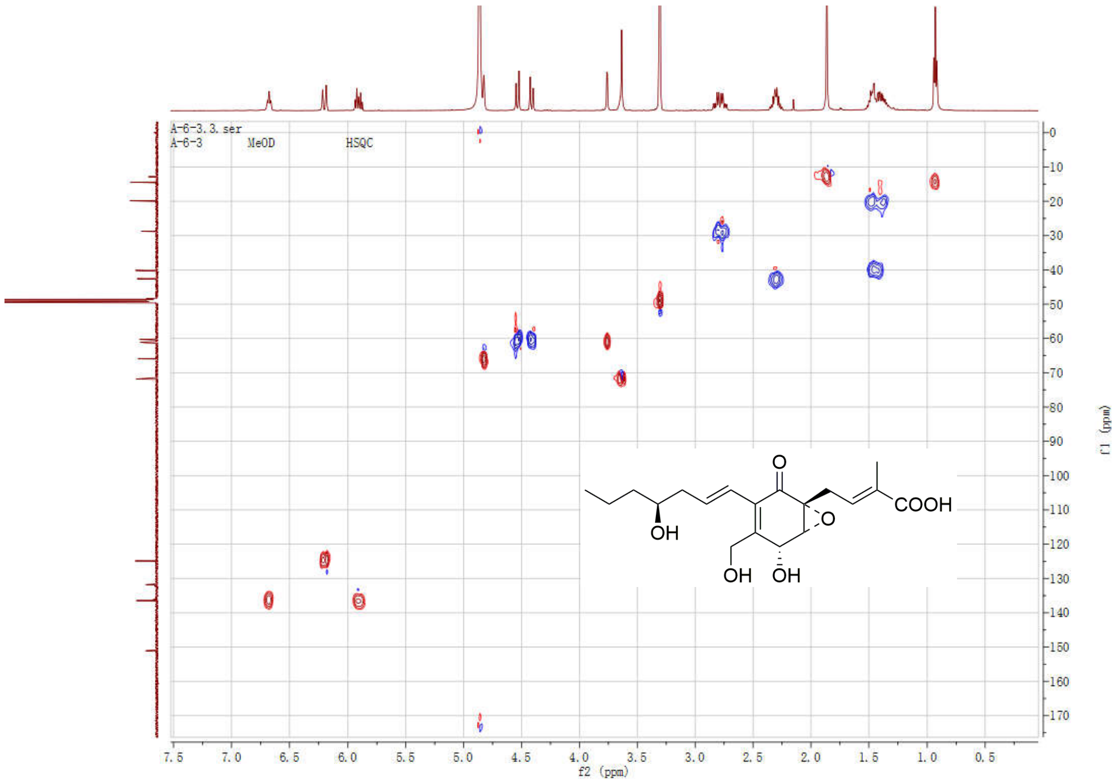

Figure S30

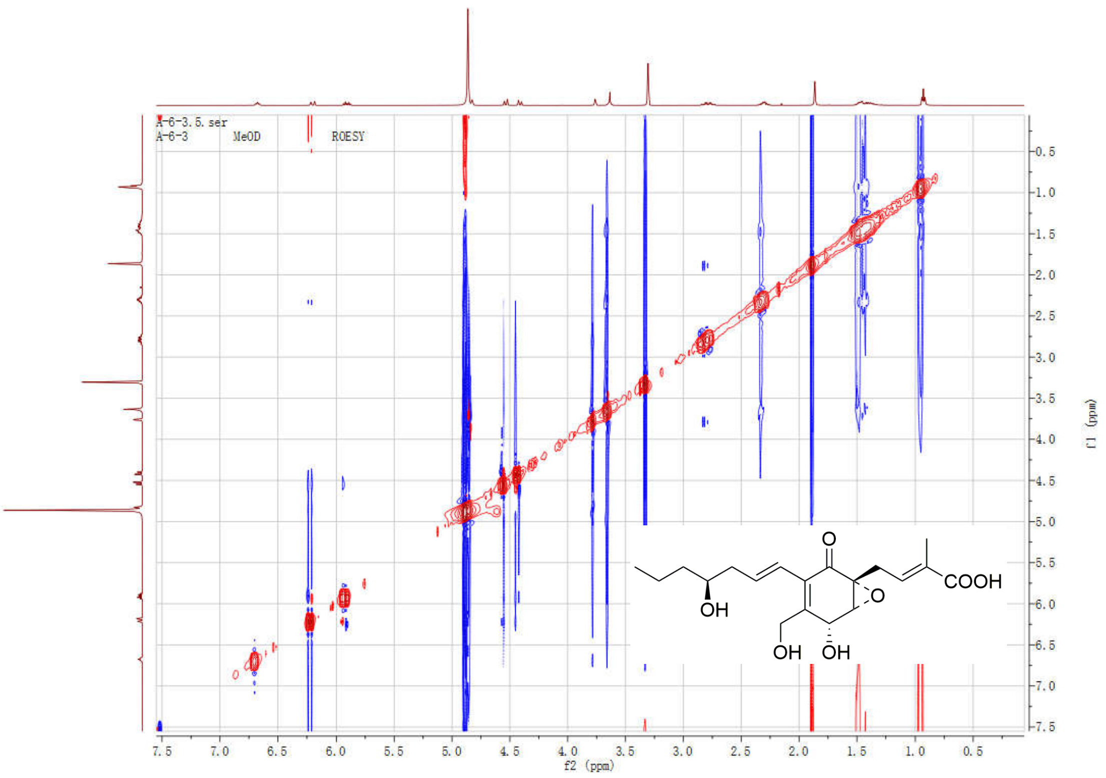

Figure S31

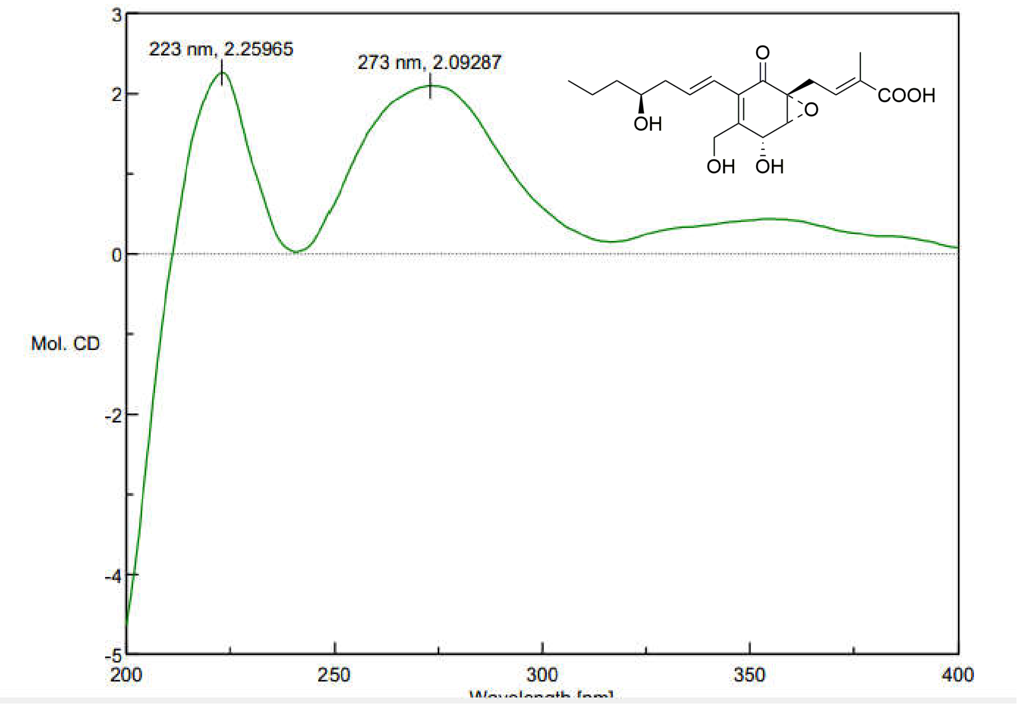

Figure S32

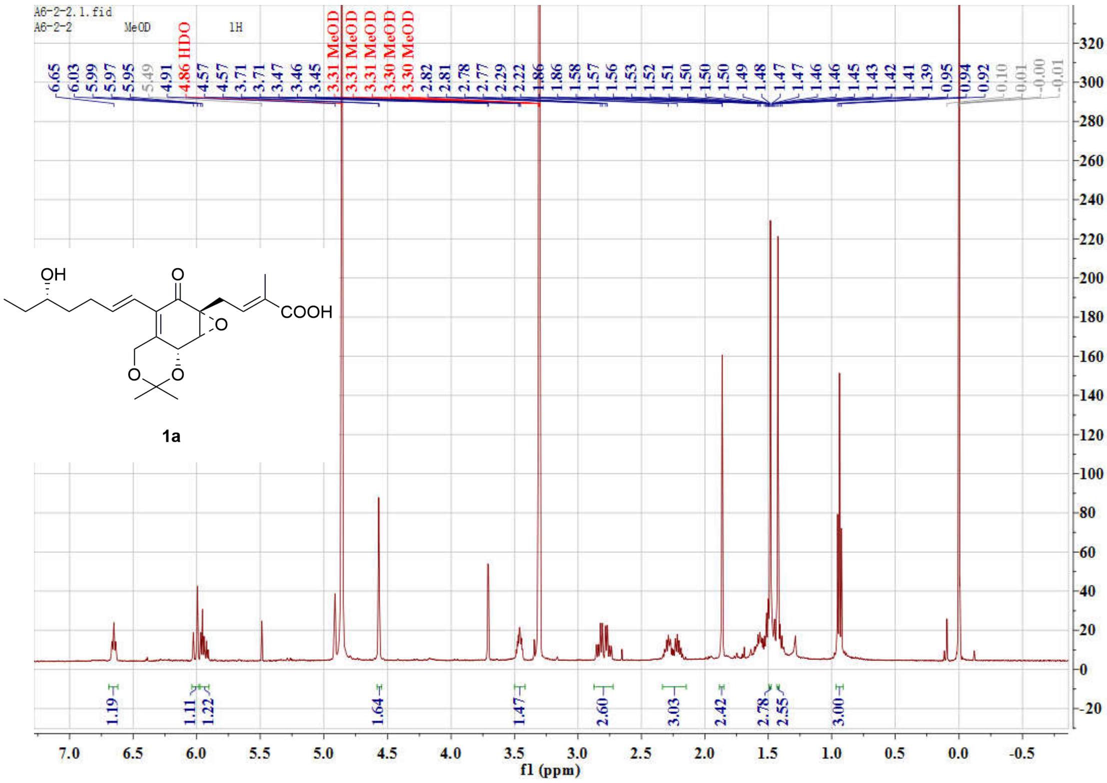

Figure S33

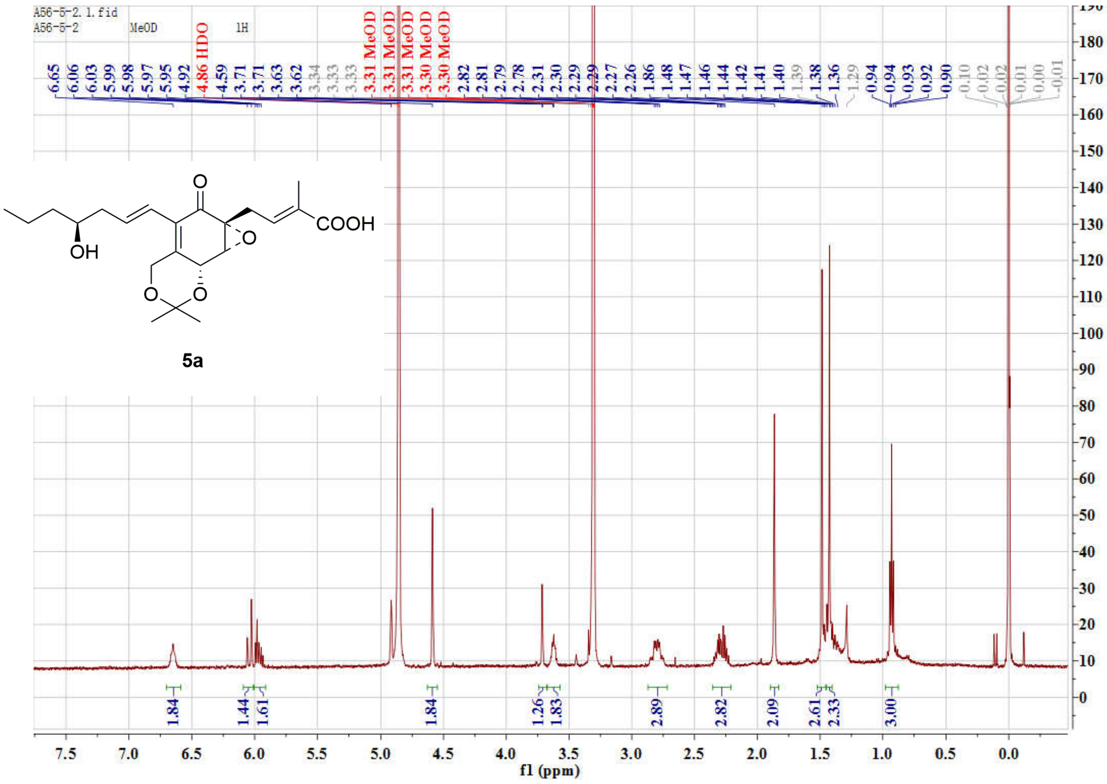

Supplement: Supplementary file 1 — NMR and CD data of new compounds 1–5, 1HNMR spectral of 1a and 5a. [file 6961928.f1.pdf]
